# Conserved proline residues in the coiled coil–OB domain linkers of Rpt proteins facilitate eukaryotic proteasome base assembly

**DOI:** 10.1016/j.jbc.2021.100660

**Published:** 2021-04-14

**Authors:** Chin Leng Cheng, Michael K. Wong, Yanjie Li, Mark Hochstrasser

**Affiliations:** 1 Department of Molecular Biophysics and Biochemistry, Yale University, New Haven, Connecticut, USA; 2 Department of Molecular, Cellular, and Developmental Biology, Yale University, New Haven, Connecticut, USA

**Keywords:** proteasome, ATPase, ubiquitin, protein aggregation, protein assembly, chaperone, AAA+, ATPases associated with diverse cellular activities, ARC, AAA+ ATPase forming ring-shaped complexes, BCA, bicinchoninic acid, BME, β-mercaptoethanol, CC, coiled coil, CP, core particle, IN, induced, OB, oligonucleotide/oligosaccharide-binding, P, pellet, PAN, proteasome-activating nucleotidase, PQC, protein quality control, RAC, RP assembly chaperone, RP, regulatory particle, S, supernatant, SD, synthetic defined, T, total protein, UN, uninduced, YPD, yeast extract-peptone-dextrose

## Abstract

The proteasome is a large protease complex that degrades many different cellular proteins. In eukaryotes, the 26S proteasome contains six different subunits of the ATPases associated with diverse cellular activities family, Rpt1–Rpt6, which form a hexameric ring as part of the base subcomplex that drives unfolding and translocation of substrates into the proteasome core. Archaeal proteasomes contain only a single Rpt-like ATPases associated with diverse cellular activities ATPase, the proteasome-activating nucleotidase, which forms a trimer of dimers. A key proteasome-activating nucleotidase proline residue (P91) forms *cis-* and *trans*-peptide bonds in successive subunits around the ring, allowing efficient dimerization through upstream coiled coils. However, the importance of the equivalent Rpt prolines for eukaryotic proteasome assembly was unknown. Here we showed that the equivalent proline is highly conserved in Rpt2, Rpt3, and Rpt5, and loosely conserved in Rpt1, in deeply divergent eukaryotes. Although in no case was a single Pro-to-Ala substitution in budding yeast strongly deleterious to growth, the *rpt5–P76A* mutation decreased levels of the protein and induced a mild proteasome assembly defect. Moreover, the *rpt2–P103A*, *rpt3–P93A*, and *rpt5–P76A* mutations all caused synthetic defects when combined with deletions of specific proteasome base assembly chaperones. The *rpt2–P103A rpt5–P76A* double mutant had uniquely strong growth defects attributable to defects in proteasome base formation. Several Rpt subunits in this mutant formed aggregates that were cleared, at least in part, by Hsp42 chaperone-mediated protein quality control. We propose that the conserved Rpt linker prolines promote efficient 26S proteasome base assembly by facilitating specific ATPase heterodimerization.

The eukaryotic 26S proteasome is a complex and highly abundant intracellular protease that comprises at least 33 different subunits; it uses the energy of ATP cleavage to unfold polyubiquitin-modified proteins and translocate them to a central chamber for proteolysis (1, 2). Proteasomes comprise a 20S core particle (CP), which forms a barrel structure with a proteolytic chamber at its center, and a 19S regulatory particle (RP) on one or both ends of the CP. The RP is made up of two major subcomplexes, the lid and base, which can assemble independently. In the lid, a deubiquitylase subunit, Rpn11, removes ubiquitin chains from substrates before their degradation. The base includes six distinct ATPases associated with diverse cellular activities (AAA+) ATPases (called Rpt1–6 in *Saccharomyces cerevisiae*) that form a heterohexameric Rpt ring in the order Rpt1-2-6-3-4-5 (2, 3, 4). The base has three additional non-ATPase subunits: Rpn1, Rpn2, and Rpn13.

Proteasome assembly must be carefully orchestrated because of the size, complexity, and abundance of this ∼2.5-MDa complex. In eukaryotes, assembly of the base is facilitated by at least four dedicated chaperones: yeast Nas2 (p27/PSMD9 in humans), Nas6 (p28/gankyrin/PSMD10), Rpn14 (PAAF1), and Hsm3 (S5b/PSMD5) (5, 6, 7, 8, 9). During base assembly, biochemical data suggest the Rpt subunits associate to form specific heterodimers along with their cognate chaperones: Hsm3–Rpt1–Rpt2 (and Rpn1), Nas2–Rpt4–Rpt5, and Nas6–Rpt3–Rpt6–Rpn14. These three “modules” then assemble into the ATPase ring. Yeast Adc17, an additional base assembly chaperone not found in humans, is thought to bind directly to Rpt6 and facilitate Rpt3–Rpt6 dimerization, particularly under stress conditions when increased amounts of proteasomes are required (10). Expression of all proteasome base chaperones is also induced upon proteotoxic stress to enhance proteasome biogenesis (11).

In archaea, by contrast, ATPase ring assembly likely proceeds independently of dedicated chaperones. Instead of six paralogous ATPase subunits, the archaeal ATPase ring comprises six copies of a single AAA+ ATPase ortholog called the proteasome-activating nucleotidase (PAN) (12, 13). The domain organization of PAN and the Rpts is conserved, beginning with an N-terminal coiled-coil (CC) domain followed by an oligonucleotide-/oligosaccharide-binding (OB) fold and the large and small domains typical of AAA+ ATPases (Fig. 1*A*) (14, 15). Similar to Rpt1, Rpt2, Rpt3, and Rpt5, PAN also contains a C-terminal HbYX (hydrophobic-Tyr-any residue) motif that engages surface pockets between the α subunits of the outer heptameric rings of the CP (16, 17).

**Figure 1:**
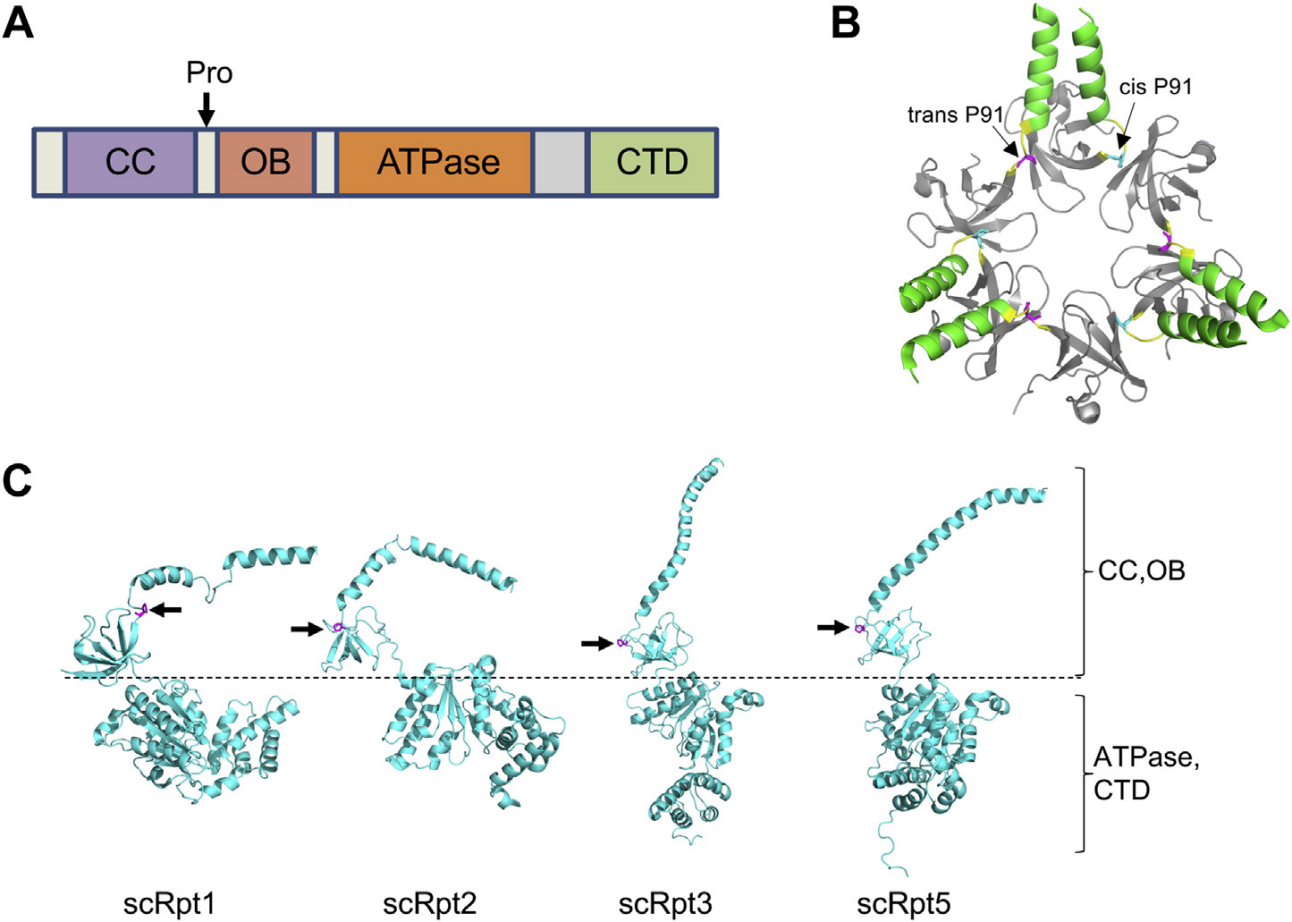
**Structures of *Methanocaldococcus jannaschii* PAN (CC and OB domains) and *Saccharomyces cerevisiae* Rpt1, Rpt2, Rpt3, and Rpt5, highlighting the position of the conserved linker proline residue.***A*, generalized domain organization of PAN and Rpt subunits. The position of the linker proline (if present) is indicated. *B*, structure of *M. jannaschii* PAN (PDB ID: 3H43). The *trans-*Pro91 residues are highlighted in *magenta* and *cis-*Pro91 in *blue*. *C*, structures of *S. cerevisiae* Rpt subunits with conserved linker prolines (*magenta* and marked with *arrows*) derived from a 26S proteasome cryo-EM structure (PDB ID: 5MP9). The *dotted line* demarcates the approximate boundary between CC–OB and ATPase domains for clearer visualization. AAA+, ATPases associated with diverse cellular activities; CC, coiled coil; CTD, C-terminal domain characteristic of AAA+ ATPases; OB, oligonucleotide-/oligosaccharide-binding domain; PAN, proteasome-activating nucleotidase; PDB, Protein Data Bank.

In *Methanocaldococcus jannaschii*, the PAN ring arranges in a trimer-of-dimers configuration (14). Crystal structures of the N-terminal CC–OB segment of the PAN revealed that the formation of dimers is dictated by the ability of the peptide bond preceding a specific proline residue, P91, in the short linker between CC and OB domains to adopt a *cis-*conformation in one subunit of the dimer and *trans-*conformation in the other (Fig. 1*B*) (14). Analysis of peptide bonds in known protein structures revealed that 6.5% of total imide bonds (X-Pro peptide bonds) have a *cis-*conformation while only 0.05% of all amide bonds (X-nonPro peptide bonds) are in a *cis-*conformation (18). The higher abundance of *cis-*isomers of proline is due to the lower energy difference between *cis-* and *trans*-isomers than other amino acids (19). Despite this small energy difference, interconversion between *cis-* and *trans-*conformations of proline is a slow process and can be rate-limiting for protein folding and unfolding (20, 21). Cells encode multiple prolyl isomerases that catalyze this interconversion (22).

An attempt to characterize recombinant *M. jannaschii* PAN–P91A and PAN–P91G mutant proteins *in vitro* was unsuccessful because the complexes were unstable, further highlighting the importance of this residue in PAN ring assembly (14). In another study, ATP-independent chaperone activity of a PAN homolog called the AAA+ ATPase forming ring-shaped complexes (ARC) from the actinobacterial species *Rhodococcus erythropolis* was investigated *in vitro*; mutation of the conserved proline (P62) in ARC-N (consisting of CC and OB domains) was found to significantly reduce the ability of the complex to inhibit aggregation of denatured citrate synthase and luciferase, suggesting that the conserved proline is important for activity (23).

The equivalent proline residue is found in Rpt1 (P96), Rpt2 (P103), Rpt3 (P93), and Rpt5 (P76) in *S. cerevisiae* (Fig. 1*C*) (24). Despite the importance of this residue in archaeal and actinobacterial proteasomal ATPases, its significance in eukaryotic Rpt subunits remains unexplored. Based on the order of the Rpt subunits in the heterohexamer and their pairwise interaction during base assembly, Rpt2, Rpt3, and Rpt5 have been predicted to have their linker prolines in the *cis*-conformation (4). Structures of 26S proteasomes determined using cryogenic electron microscopy (cryo-EM) have allowed visualization of subunit interactions within the proteasome and different conformational states (1). However, there is currently no consensus on the *cis-* and *trans-*configuration at these Rpt prolines based on available cryo-EM structures of human and yeast proteasomes, likely because of insufficient resolution in these regions.

Here, we show that, collectively, the conserved linker proline residues in Rpt2, Rpt3, and Rpt5 are important for proper proteasome base assembly in *S. cerevisiae*. Furthermore, we provide evidence for distinct contributions of the Hsp42 chaperone and the Not4 ubiquitin ligase in promoting base assembly in yeast expressing proline-to-alanine mutations in both Rpt2 and Rpt5 by suppression of the aggregation of these subunits.

## Results

### Importance of conserved Rpt linker prolines for proteasome assembly

**Figure 2:**
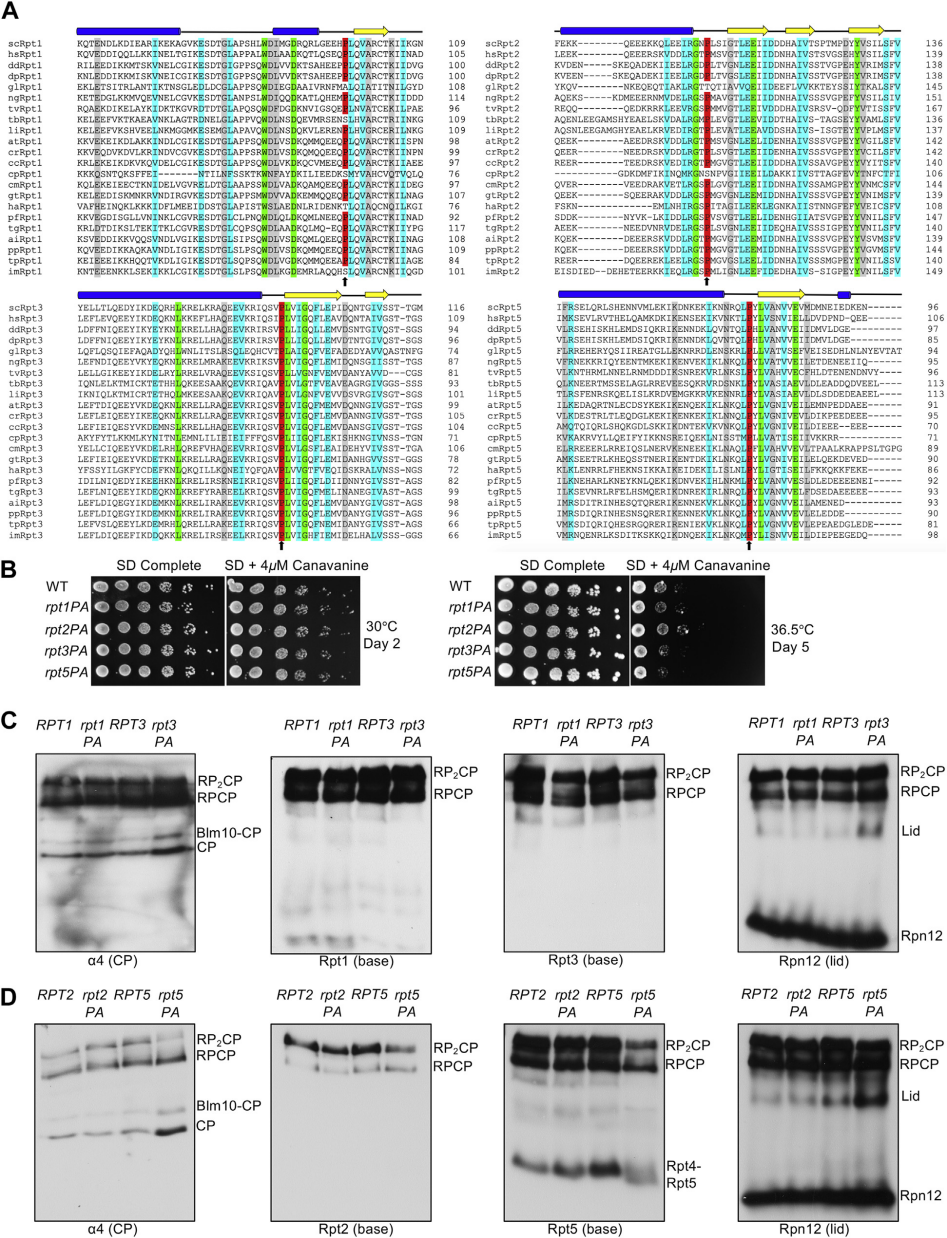
**Rpt Pro-to-Ala mutants have no growth defects but minor proteasome assembly defects.***A*, sequence alignment of Rpt1, Rpt2, Rpt3, and Rpt5 from highly diverged eukaryotic species in the region around the conserved proline residue (*arrow*). Secondary structural elements above the alignments are based on secondary structure predictions (*via* PSIPRED) of *Saccharomyces cerevisiae* subunits (*blue*, helices; *yellow arrows*, beta strands). Sequence alignments conducted with the EMBOSS (EMBL-EBI) alignment tool. (*Green*: complete conservation of residue; *cyan*: conserved residues with highly similar properties; *gray*: conserved residues with moderately similar properties). Species selected from all six recognized eukaryotic supergroups. See Table S1 for species abbreviations. *B*, growth assays of single Pro-to-Ala mutants. Yeast cultures were subjected to 6-fold serial dilutions and spotted on the indicated plates. *C*, visualization of proteasomes by immunoblot analyses of yeast *RPT1/rpt1–P96A* and *RPT3/rpt3–P93A* whole-cell extracts separated by nondenaturing PAGE. Strains were grown in selective defined media at 30 °C to the midexponential phase. RP_2_–CP and RP–CP are doubly and singly capped 26S proteasomes, respectively. *D*, visualization of proteasomes by immunoblot analyses of yeast *RPT2/rpt2–P103A* and *RPT5/rpt5–P76A* whole-cell extracts separated by nondenaturing PAGE. Strains were grown as in *panel C*. CP, core particle; RP, regulatory particle.

Based on phylogenetic analysis of proteasomal ATPases from deeply divergent eukaryotes, we found that the N-domain linker proline is strictly conserved in Rpt3 and Rpt5, highly conserved in Rpt2, and more loosely conserved in Rpt1 (Fig. 2*A*; Table S1). Because Rpt2, Rpt3, and Rpt5 belong to distinct heterodimer pairs, this finding is consistent with the hypothesis that the conserved proline residue in these subunits allows the *cis*-peptide conformation. This should kink the CC–OB linker and facilitate helix interaction and CC formation with the appropriate *trans*-Rpt partner (14, 23).

To determine the importance of these prolines *in cellulo*, we made Pro-to-Ala substitutions at Rpt1–P96, Rpt2–P103, Rpt3–P93, and Rpt5–P76 and determined their impact on yeast growth. Perhaps surprisingly, none of the resulting single mutants showed obvious growth defects compared with WT cells even under proteotoxic stress conditions (Fig. 2*B*). To investigate if these mutations affect proteasome assembly, we subjected whole-cell lysates from these strains to native gel immunoblot analyses. Despite their lack of obvious growth impairment, the *rpt3–P93A* and *rpt5–P76A* mutants exhibited defects in proteasome assembly based on excess accumulation of free lid subcomplex, CP and Blm10-CP (Blm10 is an alternative CP regulator) (Fig. 2, *C* and *D*). The *rpt5–P76A* mutant suffered a more pronounced assembly impairment based on the decreases in doubly capped 26S proteasomes and the Rpt4–Rpt5 intermediate (Fig. 2*D*). We also observed a characteristic smear or doublet near the position where the Rpt4–Rpt5 complex normally migrates (Fig. 2*D*). These species might represent either a unique Rpt5-containing assembly intermediate or a dead-end complex.

Consistent with the effects of the above Pro-to-Ala mutations on proteasome base assembly, they also caused synthetic growth defects at an elevated temperature when combined with *hsm3Δ*; loss of the Hsm3 RP assembly chaperone (RAC) has the strongest effect on growth of any single RAC mutant (Table 1; Fig. S1) (5, 6, 7, 8, 9). The *rpt2–P103A* and *rpt5–P76A* alleles also displayed synthetic defects with *nas2Δ* and *nas6Δ*, respectively. The latter each lack a RAC that promotes assembly of an Rpt heterodimer not affected directly by the respective Rpt Pro-to-Ala mutation; thus, two different base assembly modules are impacted in these mutant combinations, possibly accounting for the synthetic effects on growth. Interestingly, an *ADC17* deletion did not exhibit synthetic defects with any single *rpt–PA* single mutant although Adc17 is thought to promote Rpt3–Rpt6 heterodimerization (Table 1; Fig. S1) (10). Adc17 expression is induced under proteotoxic stress conditions (10, 11), but even under these conditions, no genetic interaction was seen (Fig. S2*A*). The *rpt–PA* single mutations might not be sufficiently detrimental to require Adc17 for enhanced base assembly. In support of this, we found that *adc17Δ* displayed a slight synthetic defect with the more deleterious *rpt2–P103A rpt5–P76A* double mutant (Fig. S2*B*). In addition, there is likely redundancy among the three chaperones affecting the Rpt3–Rpt6 module. Nas6 and Rpn14 could still promote proper assembly of Rpt3–Rpt6 in the absence of Adc17. No additional growth defects were observed when these single Rpt mutations were crossed into yeast strains with the CP assembly chaperone deletions *pba1Δ* or *pba4Δ*. Notably, *rpt1–P96A*, which affects the ATPase subunit with the least conserved linker proline, did not exhibit synthetic defects with any tested base assembly chaperone deletion (Table 1; Fig. S1).

**Table 1:** Synthetic genetic interactions between *rpt1–P96A*, *rpt2–P103A*, *rpt3–P93A*, or *rpt5–P76A* with the base and CP assembly chaperone gene deletions

Strain	*hsm3*Δ	*nas2*Δ	*nas6*Δ	*rpn14*Δ	*adc17*Δ
*rpt1–P96A*	-	-	-	-	-
*rpt2–P103A*	a	b	-	-	-
*rpt3–P93A*	a	-	-	-	-
*rpt5–P76A*	a	-	b	-	-

Strain	*pba1*Δ	*pba4*Δ
*rpt2–P103A*	-	-
*rpt5–P76A*	-	-

Yeast growth was analyzed by streak tests on yeast extract-peptone-dextrose at 36 °C. The growth defect noted is relative to congenic yeast expressing the WT *RPT* alleles in strains with the indicated assembly chaperone gene deletions.

- No observable growth defect.

a Severe growth defect.

b Moderate growth defect.

### The Rpt5 linker proline is important for Rpt5 stability and solubility

**Figure 3:**
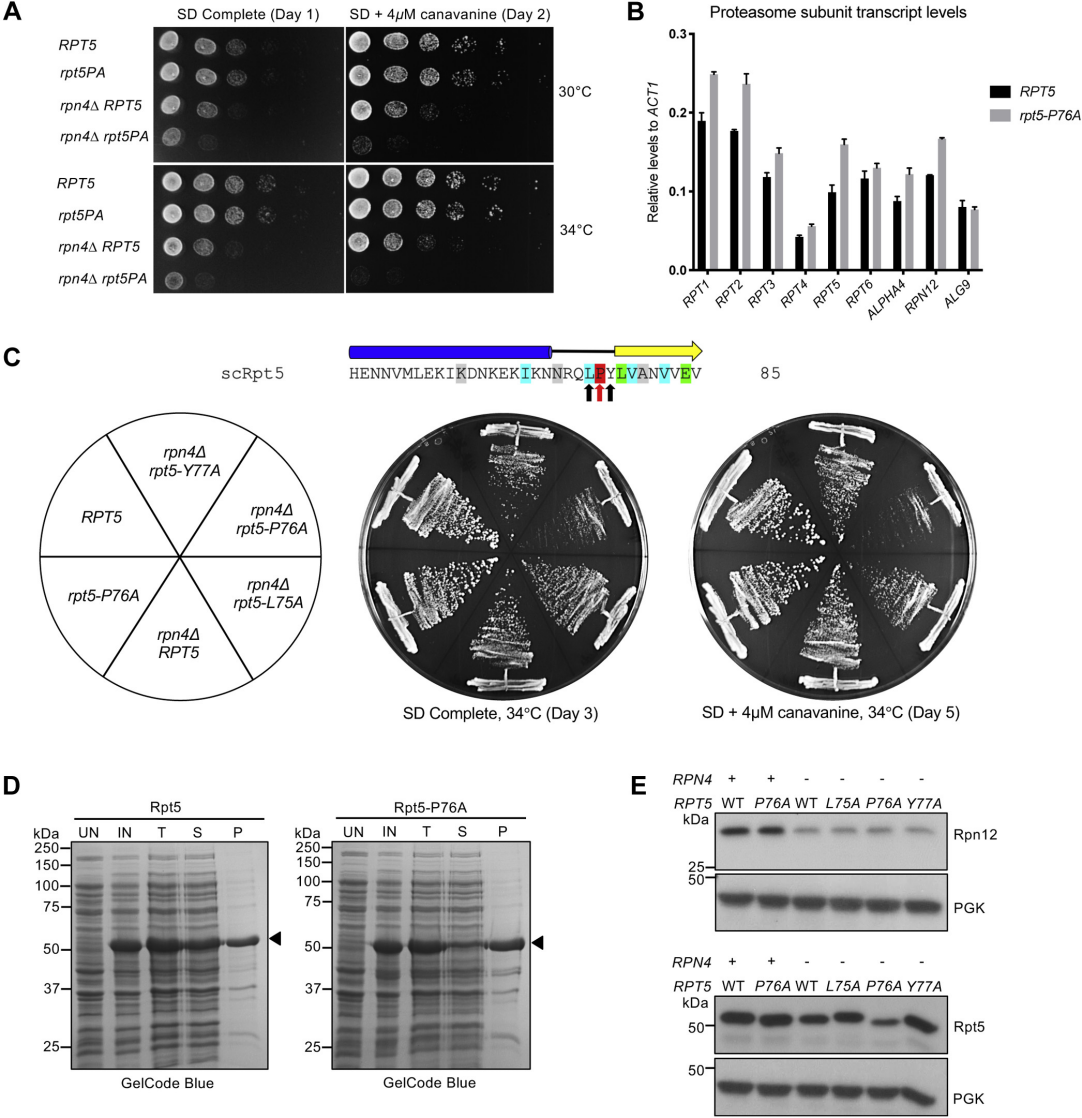
**Rpt5–P76A has unique defects among the Pro-to-Ala linker mutants.***A*, synthetic growth defects of an *rpt5–P76A rpn4*Δ double mutant. Cells were spotted as in Figure 2B. *B*, transcript levels for all proteasome subunits are consistently higher in *rpt5–P76A* than *RPT5* cells, as measured by RT-qPCR. *ALG9* serves as an internal control. (Mean SD; n = 3, technical replicates). *C*, growth of strains streaked on the indicated plates. A synthetic defect with *rpn4*Δ is seen with *rpt5–P76A* but is not observed with the flanking *rpt5–L75A* and *rpt5–Y77A* mutations. In the schematic, Rpt5–P76 is indicated with a *red arrow*, whereas the flanking residues that were mutated are indicated with *black arrows*. *D*, a higher fraction of bacterially expressed recombinant Rpt5–P76A is insoluble than that of WT Rpt5. *Arrowheads* denote WT Rpt5 or Rpt5–P76A protein. *E*, steady-state levels of soluble Rpt5–P76A are lower than that of WT Rpt5 in an *rpn4*Δ background. Yeast strains were grown in YPD at 30 °C to the midexponential phase. Phosphoglycerate kinase (PGK) served as a loading control. IN, induced; P, pellet; T, total protein; S, supernatant; UN, uninduced; YPD, yeast extract-peptone-dextrose.

Of the four individual Pro-to-Ala mutants analyzed, *rpt5–P76A* showed the strongest proteasome assembly defects, yet the mutant still appeared to grow normally. We investigated whether *rpt5–P76A* exhibited a synthetic defect when combined with a deletion of the proteasome transcription factor gene *RPN4*. Rpn4 is required for normal levels of proteasome subunit transcription and is upregulated when proteasome activity is reduced (25, 26). Indeed, deletion of *RPN4* resulted in substantial synthetic growth defects with *rpt5–P76A* (Fig. 3*A*). This was not observed when *rpn4Δ* was combined with *rpt1–P96A*, *rpt2–P103A*, or *rpt3–P93A* (Fig. S3*A*). The growth defects of *rpn4Δ rpt5–P76A* were paralleled by strongly reduced 26S proteasome formation *in vivo* (Fig. S3*B*). Quantitative RT-PCR analysis also revealed slight but consistent increases in proteasome subunit transcript levels in *rpt5–P76A* relative to *RPT5* cells, as expected if Rpn4-induced transcription was partially compensating for reduced RP base assembly in the mutant (Fig. 3*B*). When we made Ala substitutions in the two residues flanking Rpt5–P76 (Rpt5–L75A and Rpt5–Y77A), little or no synthetic defect was seen with *RPN4* deletion, demonstrating the specificity of the *rpn4Δ rpt5–P76A* interaction (Fig. 3*C*).

Next, we expressed recombinant WT Rpt5 and mutant Rpt5–P76A in *Escherichia coli* and determined the solubility of these proteins *via* a pelleting assay. For this, we lysed bacterial cells expressing each protein under nondenaturing conditions and subjected the lysates to centrifugation. The supernatant (S) fraction contained soluble proteins, and the pellet (P) fraction included aggregated proteins. Rpt5–P76A had a much higher propensity to aggregate than WT Rpt5, as most of the mutant protein was in the P (Fig. 3*D*). This observation was paralleled by the finding that steady-state levels of soluble Rpt5 were lower in *rpn4Δ rpt5–P76A* than in *rpn4Δ RPT5* yeast (Fig. 3*E*). These data suggest that Rpt5–P76A is prone to misfolding and aggregation. Rpt5–P76A aggregation is associated with proteasome assembly defects in mutant yeast cells and consequently, upregulation of proteasome subunit genes *via* Rpn4 to compensate for the depletion of the compromised mutant subunit. To investigate if there is a defect in Rpt5–P76A binding to Rpt4 in yeast, we conducted anti-FLAG immunoprecipitations using Nas2–3xFLAG expressed in *RPT5* and *rpt5–P76A* cells. As noted above, Nas2 forms a ternary assembly complex with Rpt4 and Rpt5 (5). We did not observe a difference in the ratio of Rpt4 to Rpt5 immunoprecipitated in *RPT5* as compared with *rpt5–P76A* cells (Fig. S4; see Discussion).

### Double *rpt2–P103A rpt5–P76A* mutant has synthetic assembly and growth defects

**Figure 4:**
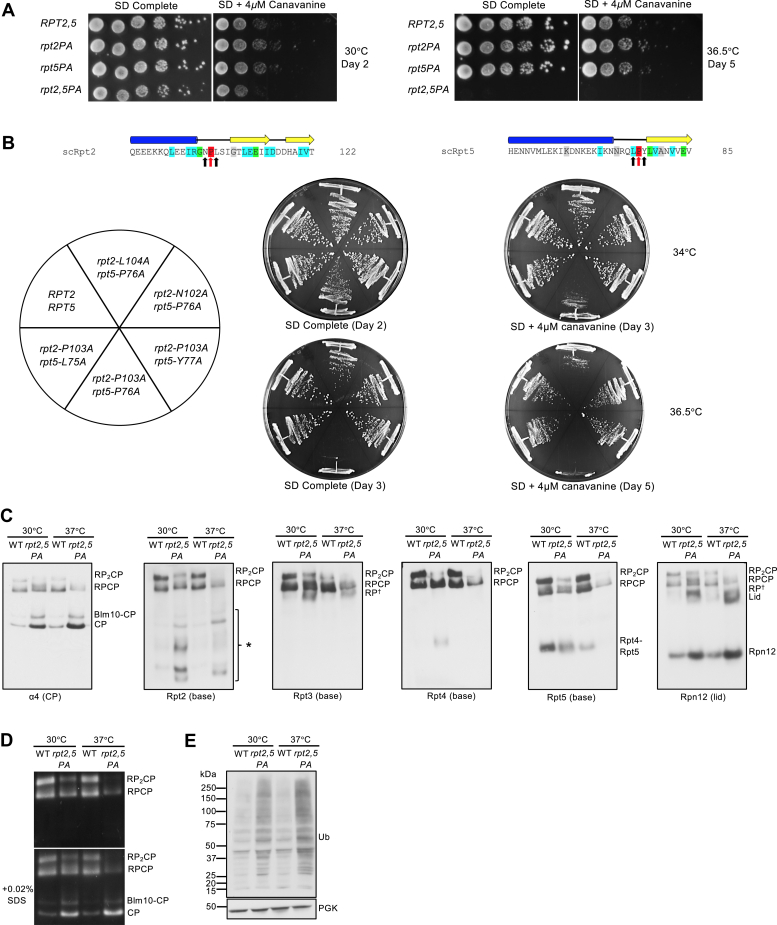
**The *rpt2–P103A rpt5–P76A* double mutant has a strong synthetic growth defect.***A*, growth assays of the *rpt2–P103A rpt5–P76A* (*rpt2,5PA*) double mutant compared with WT and single mutant cells. Cultures were spotted as in Figure 2B. *B*, growth assays highlighting the specificity of the strong *rpt2,5PA* growth defect. A milder synthetic growth defect was also observed in *rpt2–L104A rpt5–P76A* cells. *C*, visualization of proteasome complexes by immunoblot analyses of yeast *rpt2,5PA* whole-cell extracts separated by nondenaturing PAGE. Defects are more severe in cells grown at an elevated temperature. Yeast strains were grown in YPD to the midexponential phase at the indicated temperatures. The *asterisk* denotes Rpt2-containing subcomplexes. *D*, Suc-LLVY-AMC substrate overlay assay reveals lower overall proteasome activity in the *rpt2,5PA* mutant. Subsequent SDS addition to the gel allows visualization of free CP (and Blm10-CP) activity. *E*, anti-ubiquitin immunoblot analysis of yeast whole-cell lysates resolved on a denaturing gel shows accumulation of ubiquitin–protein conjugates in the *rpt2,5PA* mutant. Anti-PGK blotting was used as a loading control. CP, core particle; PGK, phosphoglycerate kinase; RP, regulatory particle; RP^†^, RP or RP-like complex; YPD, yeast extract-peptone-dextrose.

In the archaeal PAN ATPase, the single PAN–P91A mutation disrupts all subunits of the hexamer but in particular the three “*cis*” subunits, resulting in a severe defect in the ring assembly (14). We next investigated the effect on yeast growth of proline-to-alanine mutations in pairs of Rpt subunits. Of the six possible double-mutant combinations, we found that only one, *rpt2–P103A rpt5–P76A (rpt2,5PA)*, resulted in a growth defect, which was severely exacerbated at an elevated temperature (Fig. 4*A*; Fig. S5). To determine the specificity of the negative synthetic interaction between *rpt2–P103A* and *rpt5–P76A*, we tested if double-mutant combinations with mutations on both flanking residues of Rpt2–P103 and Rpt5–P76 result in similar growth defect as *rpt2,5PA*. Of all double-mutant combinations tested, only *rpt2–L104A rpt5–P76A* displayed a moderate growth defect at an elevated temperature on synthetic defined (SD) + 4 μM canavanine plates, although the defect was not nearly as severe as that of *rpt2,5PA* (Fig. 4*B*).

The *rpt2,5PA* mutant had a pronounced proteasome assembly defect characterized by accumulation of free CP, Blm10-CP, and lid subcomplex and decreased levels of singly (RPCP) and especially doubly capped (RP_2_CP) 26S proteasomes (Fig. 4*C*). In addition to these species, the double mutant also accumulated a number of novel Rpt2-containing subcomplexes, which might be dead-end complexes (Fig. 4*C*). Interestingly, these subcomplexes were also found in *hsm3Δ RPT2* cells and at higher levels in *hsm3Δ rpt2–P103A* (Fig. S6). Because the *HSM3* deletion had strong synthetic growth defects with *rpt2–P103A, rpt3–P93A,* and *rpt5–P76A* single mutants (Table 1; Fig. S1), the presence of the Hsm3 chaperone likely limits the accumulation of these subcomplexes and promotes proper base assembly in the single mutants. The *rpt2,5PA* strain also accumulated an Rpt4-containing subcomplex at 30 °C but not at 37 °C, suggesting this complex might be unstable (Fig. 4*C*). Based on anti-Rpt5 immunoblotting, the Rpt4–Rpt5 complex is also present at 30°C but not at 37 °C. This assembly intermediate may be less stable or unable to form at elevated temperatures (Fig. 4*C*). Consistent with the decrease in level of full proteasomes in *rpt2,5PA*, the mutant cells exhibited lower total proteasomal peptidase activity (Fig. 4*D*) and an increased accumulation of cellular ubiquitin conjugates (Fig. 4*E*). Defects in proteasome assembly and activity in the double mutant were worse at elevated temperatures (Fig. 4, *C*–*E*).

### Rpt5–PA ubiquitination and mutant E3 Not4–L35A suppression of *rpt2,5PA*

**Figure 5:**
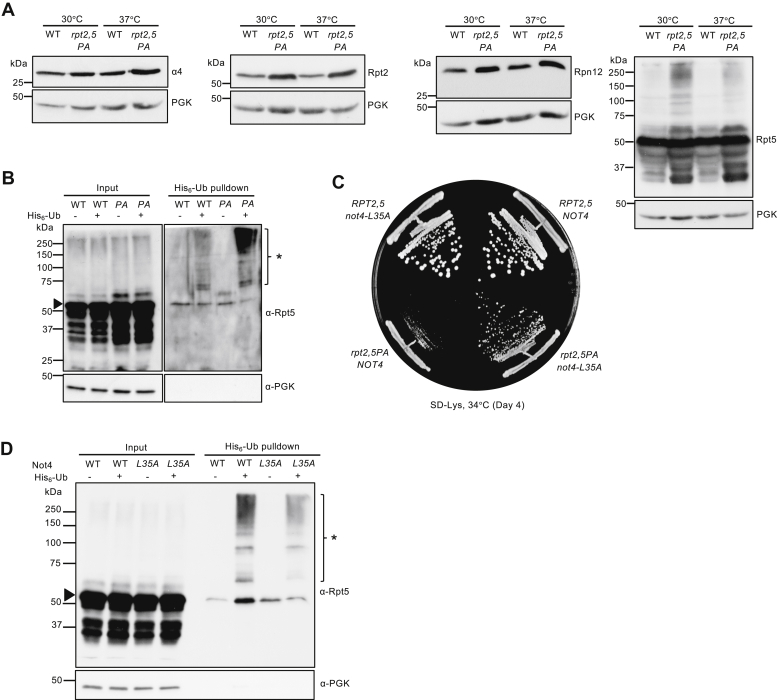
**Increased ubiquitination of Rpt5 in *rpt2,5PA* cells and partial suppression of the *rpt2,5PA* growth defect by the *not4–L35A* E3 catalytic mutation.***A*, increased proteasome subunit steady-state levels (*left three panels*) and accumulation of high-molecular-mass species of Rpt5–P76A (*rightmost panel*) in *rpt2,5PA* cells. Anti-PGK blotting was used as a control for sample loading. Yeast strains were grown in YPD at the indicated temperatures to the midexponential phase. *B*, purification of His_6_-tagged ubiquitin conjugates using a Ni-NTA resin reveals higher levels of ubiquitinated Rpt5 in *rpt2,5PA* (PA) cells, especially at a nonpermissive temperature. Eluted proteins were resolved by SDS-PAGE and immunoblotted with anti-Rpt5 antibodies. The *arrowhead* denotes unmodified Rpt5 band. The *asterisk* denotes ubiquitinated Rpt5 species. *C*, catalytically inactive Not4–L35A partially suppresses the *rpt2,5PA* growth defect. WT Not4 or Not4–L35A was expressed from a low-copy pRS317 plasmid under its native promoter in WT *RPT2,5* or mutant *rpt2,5PA* strains in which the chromosomal *NOT4* gene was deleted. *D*, inactive Not4–L35A partially suppresses the aberrant ubiquitination of Rpt5 in *rpt2,5PA* cells. Plasmids expressing His_6_-tagged or untagged ubiquitin were transformed into the *rpt2,5PA* strain expressing either WT Not4 or Not4–L35A, as in *panel C*. Purification of His_6_-tagged ubiquitin conjugates using a Ni-NTA was performed as in *panel B*. PGK, phosphoglycerate kinase; YPD, yeast extract-peptone-dextrose.

Analyses of steady-state levels of proteasome subunits revealed that the overall levels of subunits in *rpt2,5PA* did not decrease at either a permissive or nonpermissive temperature (Fig. 5*A*). In fact, overall levels of subunits increased despite the strong reduction in fully formed proteasomes in the mutant strain, hinting at a reduced elimination of defective proteasome subunits/subcomplexes. Indeed, we observed an accumulation of high-molecular-weight Rpt5-containing species that could be ubiquitinated forms of Rpt5-PA (Fig. 5*A*). To confirm this, we conducted ubiquitin pull-down assays and found higher levels of ubiquitinated Rpt5 in the *rpt2,5PA* mutant than in the WT strain (Fig. 5*B*).

It was previously reported that Rpt ring assembly is regulated through selective ubiquitination of Rpt5 by the E3 ligase Not4 (27). When ubiquitination sites on Rpt5 are exposed during assembly of the ATPases because of the absence of Hsm3 and Nas2 or during defective base assembly, Rpt5 is selectively ubiquitinated and ring assembly is inhibited (27). We speculated that Not4 similarly inhibits ATPase ring assembly in *rpt2,5PA* cells because of the presence of these mutant Rpt5 species. Indeed, when *NOT4* was replaced with the catalytic mutant *not4–L35A*, the growth defect of *rpt2,5PA* cells was partially suppressed (Fig. 5*C*). The enhanced growth correlated with a reduction in ubiquitinated Rpt5 in *rpt2,5PA not4–L35A* cells based on ubiquitin pull-down assays (Fig. 5*D*). Not4 regulation of proteasome assembly is likely specific to RP base mutants as *not4–L35A* did not suppress the temperature sensitivity of either *pre9Δ* (CP subunit) or *sem1Δ* (lid subunit) mutant (Fig. S7).

### Hsp42-mediated PQC regulates cell fitness and aggregation of Rpt2 and Rpt5

**Figure 6:**
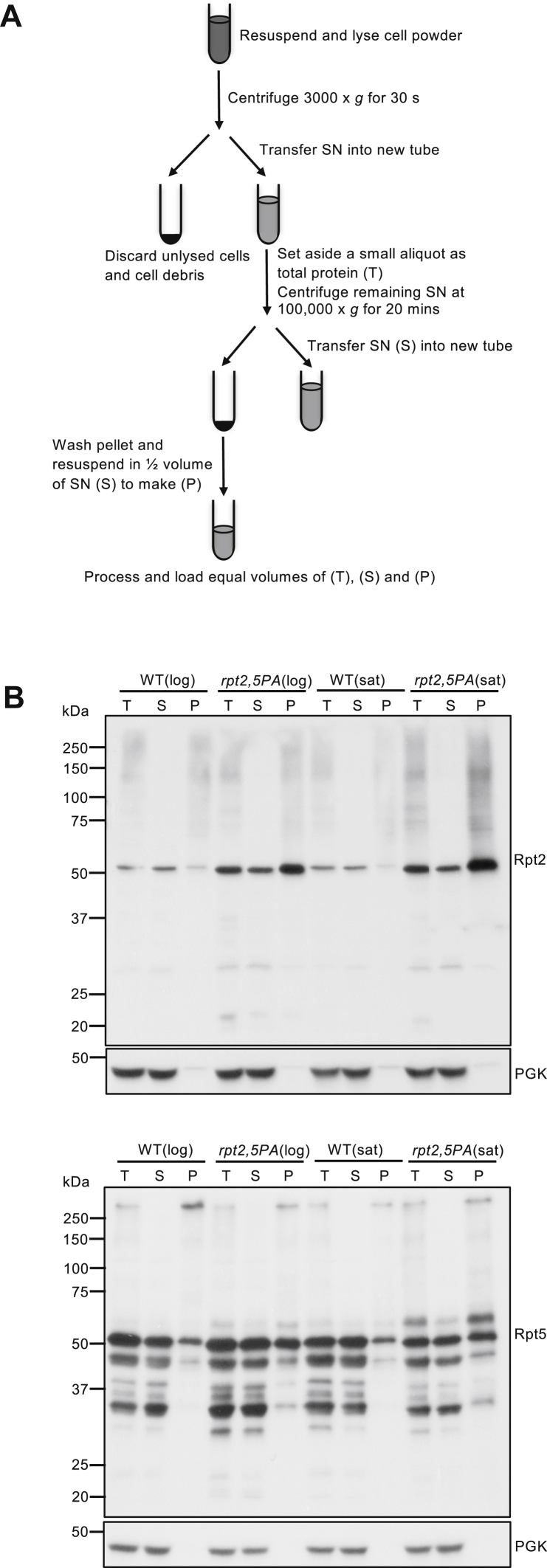
**Rpt2 and Rpt5 subunits in *rpt2,5PA* cells are prone to aggregation**. *A*, aggregation assay workflow. Yeast strains were grown in the synthetic defined (SD) medium with casamino acids at 37 °C. *B*, increased Rpt2 and Rpt5 aggregation at a high temperature as seen by an increase in these proteins in the pellet (P) compared with the supernatant (S) fraction in native extracts from *rpt2,5PA* cells. Anti-PGK blotting was used as a control for relative (soluble) protein loading. PGK, phosphoglycerate kinase; T, total protein.

Because overall levels of Rpt subunits in *rpt2,5PA* did not decrease even at elevated temperatures (Fig. 5*A*), and levels of full proteasomes and soluble Rpt intermediates/subcomplexes were further reduced at elevated temperatures (Fig. 4*C*), we speculated that Rpt subunits in this mutant have the propensity to be sequestered either for storage for future use or as insoluble aggregates for degradation or elimination *via* mother cell retention. We conducted aggregation assays using yeast whole-cell lysates to determine if the subunits form insoluble aggregates at elevated temperatures (Fig. 6*A*). We found that the double mutant had a higher P-to-S ratio for Rpt2 and Rpt5 subunits than WT, suggesting that these subunits aggregate in the mutant (Fig. 6*B*). Unlike Rpt2 and Rpt5, aggregation was not as prominent for the CP subunit α4 and was absent for lid subunit Rpn12 in the mutant strain (Fig. S8*A*). Rpt3 also aggregated in the mutant but Rpt4 did not (Fig. S8*A*). However, overall levels of Rpt4 seemed to be lower in the mutant in samples collected from saturated cultures, suggesting that Rpt4 expression might be suppressed or that it is selectively degraded (Fig. S8*A*). The smears observed predominantly above Rpt2 and Rpt3 monomers in total protein (T) and P fractions in Rpt2 and Rpt3 immunoblots are likely primarily SDS-resistant aggregates that are recognized by the Rpt2 and Rpt3 antibodies. Ubiquitin pull-down assays suggested that the smear above Rpt3 monomer does contain a small fraction of ubiquitinated Rpt3 although the levels were similar in both WT and mutant strains (Fig. S8*B*). In addition to proteasome subunits, we also examined potential aggregation of the RACs Hsm3, Nas2, Nas6, and Rpn14. Only Nas2 appeared to aggregate slightly more in *rpt2,5PA* than WT cells (Fig. S9; lanes 1–3 *versus* 7–9 in each blot).

**Figure 7:**
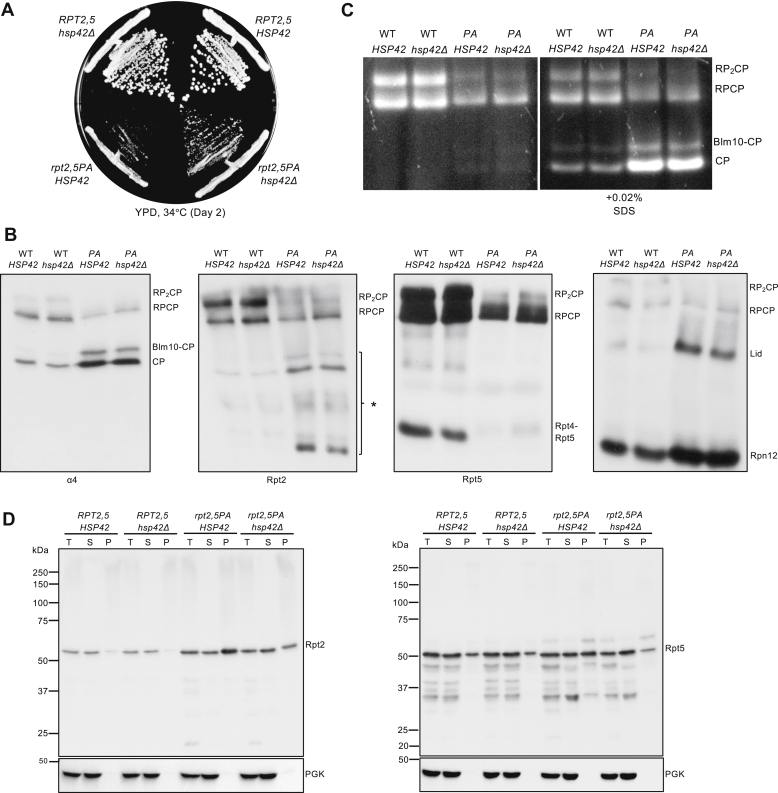
**Hsp42 promotes proteasome subunit aggregation in *rpt2,5PA* cells.***A*, *hsp42*Δ partially suppresses the growth defect of *rpt2,5PA* cells. *B*, visualization of proteasomes by immunoblotting of yeast whole-cell extracts separated by nondenaturing PAGE. Yeast strains were grown in YPD at 37 °C to the midexponential phase. *C*, Suc-LLVY-AMC overlay assay for proteasome activity. Loss of Hsp42 can very weakly suppress the proteasome assembly defect of *rpt2,5PA* cells. Yeast strains were grown as in *panel B*. *D*, aggregation assays. *HSP42* deletion in *rpt2,5PA* cells partially suppresses aggregation of Rpt2 and Rpt5. Yeast strains were grown in YPD at 37 °C to the midexponential phase. YPD, yeast extract-peptone-dextrose.

A previous study showed that a lid mutant (*rpn5ΔC*) forms aggregates at elevated temperatures, which are regulated by the protein quality control (PQC) machinery, most notably heat-shock protein Hsp42 (28). Deletion of *HSP42* in *rpn5ΔC* prevents sequestration of Rpn5*Δ*C and allows more Rpn5*Δ*C to assemble into full proteasomes, thereby strongly suppressing the growth defect of the *rpn5ΔC* mutant (28). We asked if *rpt2,5PA* is similarly regulated by Hsp42. Loss of Hsp42 partially suppressed the *rpt2,5PA* growth defect, albeit to a much lower extent than that of *rpn5ΔC* (Fig. 7*A*) (28). This partial rescue is consistent with the very modest increase in full proteasome levels when *HSP42* is deleted in *rpt2,5PA* based on native immunoblot analyses and in-gel proteasome activity assays (Fig. 7, *B* and *C*). Levels of Rpt2-containing intermediates also decreased slightly but reproducibly in *hsp42Δ rpt2,5PA* relative to *rpt2,5PA* cells, suggesting either fewer of these potential dead-end complexes form or, if functional, greater incorporation into higher-order complexes (Fig. 7*B*). We also observed a small increase in the Rpt4–Rpt5 complexes in *hsp42Δ rpt2,5PA* cells along with reduced free lid accumulation, consistent with weakly enhanced proteasome RP assembly (Fig. 7*B*). Aggregation assays showed slightly reduced aggregation of Rpt2, Rpt5, and possibly also the Nas2 chaperone, which would be consistent with enhanced formation of the Nas2–Rpt4–Rpt5 assembly module (Figs. 7*D* and S9). Interestingly, we found that *HSP42* deletion also partially rescued the temperature sensitivity of other base (*cim3-1* and *rpt4–G106D*) and CP (*pre9Δ*) mutants (Fig. S10). These findings suggest that Hsp42-mediated PQC can limit proteasome base (and CP) assembly when assembly is perturbed, analogous to its effects on mutant lid assembly.

**Figure 8:**
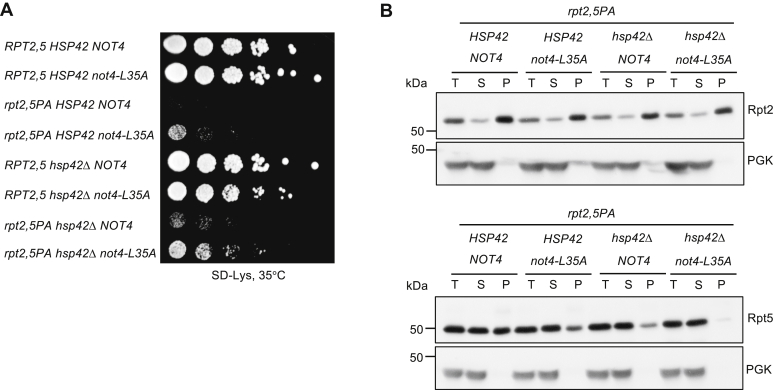
**Simultaneous loss of Hsp42 and Not4 E3 ligase activity further enhances growth of *rpt2,5PA* mutant cells.***A*, the *rpt2,5PA hsp42*Δ *not4–L35A* mutant grows better than either *rpt2,5PA hsp42*Δ or *rpt2,5PA not4–L35A*. Serial dilutions of cultures were spotted as in Figure 2*B*. *B*, the *rpt2,5PA hsp42*Δ *not4–L35A* mutant reduces aggregated Rpt5 relative to *rpt2,5PA hsp42*Δ or *rpt2,5PA not4–L35A.* Yeast strains were grown in YPD at 37 °C to the exponential phase. YPD, yeast extract-peptone-dextrose.

To investigate the epistatic relationship between the enhanced growth caused by inactivation of Not4 E3 ligase activity (Fig. 5) or Hsp42 (Fig. 7) in the *rpt2,5PA* mutant, we created an *rpt2,5PA* strain with both Not4 and Hsp42 inactivated and compared its growth with *rpt2,5PA* strains with mutation of either Not4 or Hsp42 alone. Inactivation of both Hsp42 and Not4 simultaneously further enhanced the growth of *rpt2,5PA* cells (Fig. 8*A*). This growth enhancement is consistent with a further reduction in aggregated Rpt5 in the P (Fig. 8*B*). Hence, these factors appear to modulate proteasome assembly by at least partly separate pathways.

## Discussion

Here we have shown that the conserved Rpt linker prolines promote eukaryotic 26S proteasome base assembly, most likely by facilitating specific pairwise ATPase heterodimerization. Based on structural data from their prokaryotic counterparts, this is predicted to be due to the enhanced ability of proline residues to form *cis*-peptide bonds, which would create a kink in the linker that allows the upstream helical domain of the subunit to form a CC more readily with its (*trans*) ATPase partner (14, 23). Interestingly, recent work has suggested another mechanism for promoting specific Rpt heterodimer interaction that involves pausing of the ribosome during translation of the Rpt1 and Rpt2 nascent complexes, to allow their cotranslational assembly (29). The disordered N-terminal segment of Rpt1 was shown to be important for pausing, and the Not1 subunit of the Ccr4–Not complex (which includes Not4) participates in colocalizing the stalled translation complexes. All three Rpt heterodimers also interact with dedicated (and nonhomologous) RACs; for the yeast Rpt3–Rpt6 dimer, three different RACs help promote its assembly (5, 6, 7, 8, 9, 10). Hence, eukaryotes have evolved both intrinsic and extrinsic mechanisms to increase the assembly efficiency and fidelity of the proteasomal heterohexameric ATPase ring.

### Linker proline mutations are tolerated to varying degrees

Yeast cells have a surprisingly high tolerance for single Rpt proline-to-alanine mutations based on growth analysis (Fig. 2*B*). For most of the mutants, proteasome assembly appears normal or nearly so. Despite the lack of a growth defect, the *rpt5–P76A* mutant did display a modest proteasome assembly deficiency (Fig. 2*D*). The lower solubility of Rpt5–P76A is reminiscent of the aggregation seen with the homologous archaeal PAN–P91A mutation (14).

Nas2 coprecipitation analysis revealed similar ratios of Rpt4 and Rpt5 in *rpt5–P76A* and *RPT5* strains, which would suggest that Rpt4–Rpt5 CC formation is not impaired by the *rpt5–P76A* mutation (Fig. S4). There are several caveats to this interpretation, however. First, because Nas2 binds to the C-terminal domain of Rpt5, it might not be able to distinguish Rpt4–Rpt5 dimers with improperly formed N-terminal CC domains (30). Second, it is possible that the Rpn4 transcription feedback loop increases total levels of Rpt5–P76A protein to compensate for its lower solubility and/or binding to Rpt4 (Fig. 3*B*). It is difficult to disentangle reduced solubility of Rpt5–P76A protein and defective heterodimerization with Rpt4 as the primary cause of the *rpt5–P76A* base assembly defect because they are related. Quantitative binding analysis with separately purified Rpt4 and Rpt5 will be required to determine if Rpt5–P76A has lower affinity toward Rpt4, although our finding that recombinant Rpt5 expressed in *E. coli* forms homo-oligomers would complicate such an analysis (unpublished observations).

Assembly of an Rpt heterohexamer in eukaryotes, unlike a homohexamer, may limit growth and assembly defects because of single Pro-to-Ala mutations; introduction of a single PAN–P91A mutation, by contrast, effectively disrupts all three “*cis*” subunits of the archaeal homohexamer. Consistent with such a quantitative effect, base assembly is far more severely disrupted in the double *rpt2–P103A rpt5–P76A* mutant (Fig. 4). Other factors may also contribute to tolerance of linker proline mutations in eukaryotic ATPase CC pairings. Rpt1 and Rpt2 both contain an apparently unstructured segment that bisects the CC (Fig. 1*C*). This may provide additional flexibility in CC formation between Rpt1 and Rpt2. We note the presence of a glycine residue in place of proline in the Rpt4 and Rpt6 “*trans*” subunits, which may also facilitate correct CC formation in their respective heterodimers. The glycine in Rpt4 (G104) is particularly well conserved.

The presence of certain RACs can also limit defects because of the Rpt Pro-to-Ala mutations. The *rpt2–P103A*, *rpt3–P93A*, and *rpt5–P76A* mutations have strong synthetic defects with *hsm3Δ* and with other select RAC gene deletions (Table 1; Fig. S1). Deletion of *HSM3* in an *rpt2–P103A* background also led to increased accumulation of multiple Rpt2-containing subcomplexes. These species were observed in the *rpt2,5PA* double mutant as well. The results indicate that Hsm3 plays an important role in promoting proper base assembly when these conserved proline residues are mutated (Fig. S6). By contrast, *rpt1–P96A* did not display synthetic defects with any RAC deletions. Rpt2, Rpt3, and Rpt5 belong to distinct dimer pairs in early base assembly, and their linker prolines are more highly conserved than in Rpt1. Our findings are consistent with the hypothesis that Rpt2, Rpt3, and Rpt5 are the “*cis*” subunits in the eukaryotic Rpt ring, a prediction that still awaits structural confirmation.

### Synthetic defects in *rpt2,5PA* mutant linked to severely impaired base assembly

The *rpt2,5PA* strain is the only double Pro-to-Ala mutant that displayed strong growth and proteasome assembly defects (Fig. 4). We speculate that the peptide bonds preceding these prolines have to be in a *cis*-conformation for Rpt2 and Rpt5 to associate efficiently with their partner ATPase subunits during assembly into higher-order base subcomplexes. Rpt2–P103A and Rpt5–P76A mutations may, for example, inhibit CC formation between the correct pairs of Rpt subunits and thereby disrupt proper base assembly. The tendency of Rpt5–P76A to aggregate may further enhance base assembly defects in the double mutant. A reduced ability of Rpt2- and Rpt5-containing complexes to associate properly could therefore lead to the formation of Rpt2-containing dead-end complexes or assembly intermediates, which could explain the presence of several Rpt2-containing complexes that are absent or rare in WT strains (Fig. 4*C*). The reduced accumulation of these unique complexes without an increase in higher-order complexes and full proteasomes at elevated temperature further suggests that these complexes are unstable and subsequently degraded and/or sequestered into aggregates.

High levels of ubiquitinated Rpt5–P76A also accumulate in the *rpt2,5PA* strain, suggesting that the misassembled ATPase subunit is marked for ubiquitin-dependent degradation. Expression of the Not4–L35A ubiquitin ligase catalytic mutant partially suppresses the growth defect and levels of ubiquitinated Rpt5–P76A in *rpt2,5PA* mutant, consistent with the presence of a base assembly defect and a role for Not4 in regulating base assembly in this mutant (Fig. 5) (27). The modest suppression observed relative to the base assembly mutants studied by Fu *et al*. (27) could be due to base assembly defects in *rpt2,5PA* cells that go beyond simply exposing Not4 ubiquitination sites on Rpt5; inhibition of Not4 catalytic ligase activity might therefore be insufficient for strong enhancement of base assembly in this mutant.

### Hsp42 participates in the aggregation of proteasome base subunits in *rpt2,5PA* cells

Rpt2 and Rpt5 subunits are found in aggregates in the *rpt2,5PA* double mutant (Fig. 6*B*). We attempted to tag the N termini of Rpt2 and Rpt5 with GFP to track these aggregates *via* microscopy, but the resulting strains were inviable. Nevertheless, we found that deletion of *HSP42* partially suppressed growth defects of *rpt2,5PA*, although not to the extent seen with the lid mutant *rpn5ΔC* (Fig. 7*A*) (28). The (partial) suppression of Rpt2–Rpt5 subunit aggregation, growth impairment, and proteasome assembly defects by *hsp42Δ* suggests that the Hsp42-based PQC machinery is important for regulating proteasome sequestration or PQC in different mutants of the proteasome, not just lid mutants (Fig. 7, *A* and *B*). Indeed, our data indicate that Hsp42 is a general regulator of proteasome assembly, as revealed by the ability of *hsp42Δ* to also suppress other RP base (*cim3-1* and *rpt4–G106D*) mutants and a CP (*pre9Δ*) mutant (Fig. S10). Interestingly, *hsp42Δ* did not suppress another lid mutant, *sem1Δ*, possibly because Sem1 is involved not only in proteasome assembly (31) but also in the functioning of the mature 26S proteasome (32, 33, 34) as well as that of other protein complexes (35, 36). Notably, simultaneous inactivation of both Hsp42 and Not4 E3 ligase activity further suppressed the growth defect of *rpt2,5PA* (Fig. 8*A*). This finding provides evidence for two distinct but complementary pathways that regulate the assembly of the proteasome base, at least when components of the base are defective.

In summary, our data are consistent with the hypothesis that the highly conserved Rpt linker prolines promote formation of *cis*-peptide bonds specifically in one subunit of each eukaryotic Rpt heterodimer, which facilitates their dimerization with the correct “*trans*” subunits, presumably through enhanced CC formation. Yeast cells have multiple mechanisms that allow them to tolerate mutations in the highly conserved linker prolines of the three predicted “*cis*” subunits Rpt2, Rpt3, and Rpt5. These include the Rpn4-dependent transcriptional feedback loop and Rpt heterodimer-specific assembly chaperones. On the other hand, PQC mechanisms that result in ubiquitination and degradation of mutant or misassembled subunits of the proteasome or the sequestration of aberrant assembly intermediates into Hsp42-dependent aggregates such as insoluble protein deposits enhance growth deficiencies of these mutants. Together with the recent description of cotranslational assembly of Rpt heterodimers, our results point to the importance of multiple mechanisms, which are likely intertwined, to ensure efficient and high-fidelity assembly of the eukaryotic 26S proteasome.

## Experimental procedures

### Yeast strains

Yeast strains were made following standard procedures (37). Yeast haploid strains with WT or mutant Pro-to-Ala Rpt subunit genes expressed from low-copy plasmids under their native promoters were created in strains with the corresponding chromosomal copy or copies replaced with an *HIS3* cassette as described previously (4). Because all *RPT* genes are essential, the parental strains all initially had the relevant WT *RPT* gene(s) on plasmids bearing a *URA3* selectable marker. Strains were then transformed with plasmids carrying either a *TRP1* or *LEU2* selectable marker and expressing either WT or mutant *rpt* alleles. The resulting strains were then cured of the original *URA3* plasmid by counterselection on 5-fluoroorotic acid. The list of yeast strains and plasmids used can be found in Tables S2 and S3, respectively.

### Yeast growth assays

Yeast strains were grown in a yeast extract-peptone-dextrose (YPD)-rich medium or SD media to saturation overnight. The next day, strains were diluted in sterile water at 0.2 units of absorbance at 600 nm in a final volume of 1 ml. Samples were then spotted in a 6-fold dilution series on the appropriate plates and incubated at various temperatures, and growth was monitored over several days.

### Nondenaturing gel analyses of proteasomes in whole-cell extracts

Yeast extracts for nondenaturing gel analyses were prepared as previously described with slight modifications (38). Yeast cultures were grown in YPD or SD media overnight. The next day, cultures were diluted to absorbance at 600 nm = 0.2 in YPD or SD media and grown to the midexponential phase (unless otherwise stated), washed with ice-cold sterile water, and subsequently frozen in liquid nitrogen and stored at −80 °C. Frozen cells in liquid N_2_ were ground using a mortar and pestle until a fine powder formed. The resulting powder was collected in a pre-chilled tube and incubated in proteasome extraction buffer (50 mM Tris HCl pH 7.5, 5 mM MgCl_2_, 10% glycerol, 5 mM ATP) for 10 min with occasional vortexing. Samples were then centrifuged at 22,000*g* for 10 min to remove unlysed cells and cell debris. The resulting S were collected, and the protein concentration was determined using the bicinchoninic acid (BCA) assay conducted according to manufacturer's instructions (Thermo Fisher Scientific). Fifty microgram samples were electrophoresed in 4% nondenaturing polyacrylamide gels (native PAGE). Gels were either overlayed with a fluorogenic substrate, Suc-LLVY-AMC (Sigma-Aldrich) or were used in immunoblot analyses. Details of the experimental procedures for the in-gel peptidase assay are as described (39). For analyses of steady-state levels of soluble protein from these lysates (as in Fig. 3*E*), 10 μg of each S was run in denaturing SDS gels and subjected to immunoblotting.

### Denaturing gel analyses of overall levels of proteins in yeast extracts

Yeast extracts for denaturing gel analyses were prepared as previously described with slight modifications (40). Yeast cultures were grown as above (unless otherwise noted). Absorbance at 600 nm of 2.5 units of cells was performed; those cells were harvested by centrifugation and washed with ice-cold sterile water. Samples were then resuspended in 200-μL sterile water followed by the addition of 200-μL 0.2 M NaOH and incubated at room temperature for 5 min with occasional vortexing. Cells were pelleted at 10,000*g* for 1 min, and S were discarded. Pelleted cells were resuspended in 1× SDS-PAGE sample buffer containing 4% β-mercaptoethanol (BME) and heated at 100 °C for 5 min followed by centrifugation at 10,000*g* for 1 min; 10 to 15 μL of the S was resolved in discontinuous SDS gels and subjected to immunoblot analyses.

### Aggregation assay of recombinant 6His–Rpt5 expressed in *E. coli*

Competent Rosetta DE3 cells transformed with either pET15b–6His–Rpt5 or pET15b–6His–Rpt5–P76A plasmid were grown in LB + 100 μg/ml ampicillin media overnight at 37 °C. Cultures were diluted 1:100 in fresh LB + ampicillin medium and grown to absorbance at 600 nm = 0.6 to 0.8. One unit of culture at absorbance at 600 nm was removed as an uninduced (UN) sample. Cultures were then induced with 0.2 mM IPTG (final) and grown at 16 °C overnight. One unit of culture at absorbance at 600 nm was harvested as an induced (IN) sample. Another 1.5 ml aliquot from each culture was harvested for aggregation assays and resuspended in 700 μL lysis buffer (50 mM Tris HCl pH 7.5, 150 mM NaCl, 10% glycerol, 100 μg/ml lysozyme, 1 mM PMSF) and incubated at 4 °C for 30 min. Samples were then sonicated 6 × 10 s with 10-s incubations on ice between each sonication round; 100-μL aliquot from each sample was transferred into a new tube and represented T. The remaining samples (600 μL each) were centrifuged at 21,000*g* for 5 min at 4 °C. The S was transferred into a new tube. The P was washed once with 600-μL lysis buffer and recentrifuged as above; the lysis buffer was removed, and the P was resuspended in 600-μL lysis buffer. UN and IN cell P were resuspended in 150 μL of 1× SDS sample buffer containing 1% BME. T, S, and P samples were brought to 1× concentration of SDS sample buffer containing 1% BME (final). All samples were heated at 100 °C for 5 min followed by centrifugation at 10,000*g* for 1 min. Fifteen microliter of UN and IN and 30 μL of T, P, and S were resolved in 10% denaturing gels, and the gels were stained with GelCode Blue Stain Reagent (Thermo Fisher Scientific) and imaged.

### Aggregation assays of proteasome subunits in yeast

Yeast cultures were grown in YPD or SD media (with casamino acids) overnight. The next day, cultures were diluted to absorbance at 600 nm = 0.2 in YPD or SD media (with casamino acids) and grown to the midexponential or saturation phase. Cells were harvested and washed with sterile cold water and flash-frozen in liquid N_2_. Cells were ground using a mortar and pestle until a fine powder was formed. Cell powder was resuspended in ice-cold lysis buffer (50 mM Tris pH 7.5, 150 mM NaCl, 1% glycerol, 1 mM EDTA, 1 mM PMSF, 1× EDTA-free cOmplete Protease Inhibitor Cocktail (Roche)) and vortexed intermittently during a 10-min incubation on ice. Samples were centrifuged at 3000*g* for 30 s to remove cell debris.

S were transferred to fresh tubes. BCA assays were conducted to determine T concentrations. Protein concentration was normalized across all samples tested. A small aliquot was set aside as T. The remaining normalized S were centrifuged at 100,000*g* for 20 min at 4 °C in a Beckman Coulter TLA-55 rotor. S were transferred to fresh tubes. P were then washed with the lysis buffer and recentrifuged as above. S were discarded, and the resulting P were resuspended in half the volume of the S to make the P fraction. T, S, and P samples were brought to 1× concentration of SDS sample buffer containing 1% BME (final). Samples were heated at 100 °C for 5 min followed by centrifugation at 10,000*g* for 1 min. Equal volumes of T, S, and P samples were loaded onto SDS gels and subjected to immunoblot analyses. The P sample loaded was therefore twice as concentrated as those of T and S samples to account for potentially low levels of proteins in P that might be difficult to detect.

### Antibodies and immunoblotting

After samples were resolved in denaturing SDS or nondenaturing polyacrylamide gels, proteins in the gels were transferred to PVDF membranes (Millipore). Immunoblots were analyzed using primary antibodies against α4/Pre6 (D. Wolf); Rpt1 (W. Tansey); Rpt2 (Enzo Life Sciences); Rpt3 (Enzo Life Sciences); Rpt4 (W. Tansey); Rpt5 (Enzo Life Sciences); Rpn2 (M. Glickman); Rpn12 (D. Finley); the RACs Hsm3, Nas2, Nas6, and Rpn14 (all Hochstrasser lab stocks (5)); ubiquitin (Dako); phosphoglycerate kinase (Invitrogen); and glucose-6-phosphate dehydrogenase (Sigma-Aldrich). For enhanced chemiluminescence detection, horseradish peroxidase–linked anti-mouse immunoglobulin G (from sheep) and horseradish peroxidase–linked anti-rabbit immunoglobulin G (from donkey) (both GE Healthcare) were used as secondary antibodies.

### Analyses of mRNA levels in yeast extracts

Yeast cultures were grown in SD media overnight. Cultures were diluted to absorbance at 600 nm = 0.2 in SD media and grown to the midexponential phase. Cells corresponding to one unit of absorbance at 600 nm were harvested and washed with sterile ice-cold water. Total RNA was extracted from the cells using an RNeasy Mini Kit (Qiagen) and eluted in 50-μL nuclease-free water. Contaminating DNA was subsequently removed from the samples using the DNA-*free* Kit (Ambion). Two micrograms of total RNA was reverse-transcribed using the iScript cDNA Synthesis Kit (Bio-Rad). The resulting cDNA was subjected to quantitative PCRs using iQ SYBR Green Supermix (Bio-Rad) and analyzed on a LightCycler 480 (Roche). Each quantitative PCR was conducted in three technical triplicates. All experiments were conducted according to manufacturers' instructions.

### Ubiquitin pull-down assays

To determine if proteasome subunits were ubiquitinated at nonpermissive temperatures, we conducted ubiquitin pull-down assays with slight modifications from a previously outlined protocol (41). For the experiment shown in Figure 5*B*, we transformed WT and *rpt2,5PA* strains with pUB175 (expressing untagged ubiquitin) or pUB221 (expressing His_6_-tagged ubiquitin). The yeast ubiquitin genes in these plasmids are expressed under the control of a copper-inducible *CUP1* promoter. Overnight cultures grown at 30 °C were diluted to absorbance at 600 nm = 0.2 and grown in 175-ml SD-URA for 2.5 h at 37 °C. For the experiment shown in Figure 5*D*, *rpt2,5PA NOT4* and *rpt2,5PA not4-L35A* cells were transformed with pUB175 or pUB221. Overnight cultures grown at 30 °C were diluted to absorbance at 600 nm = 0.2 and grown in 175-ml SD-URA-LYS for 2.5 h at 30 °C. Cultures were then induced with 0.5 mM CuSO_4_ (final) and grown for another 4 h. Five microliters of each culture was harvested, washed with sterile water, and saved.

The remaining culture fractions were harvested by centrifugation, washed with sterile water, and resuspended in 2-ml buffer A (6 M guanidine HCl, 0.1 mM Na_2_HPO_4_/NaH_2_PO_4_, 10 mM imidazole, pH 8.0) followed by cell disruption with glass beads for 6 × 20 s using a vortexer at top speed with 30-s breaks on ice between each round. Samples were centrifuged at 1690*g* for 15 min to remove cell debris. S were collected, and the T concentration of each sample was determined *via* Bradford assay (Bio-Rad). T was normalized to 2 mg across all samples tested and incubated with 0.25 ml of 50% Ni-NTA resin (Qiagen) for 2 h. The resin was subsequently pelleted, and the S was aspirated off. The remaining beads were washed three times with 1-ml buffer A followed by three washes with 1-ml buffer A/TI (1 volume buffer A and three volumes buffer TI–25 mM Tris HCl, 20 mM imidazole, pH 6.8) and finally once with 1-ml buffer TI. The beads were resuspended in 0.20-ml 2× SDS sample buffer (containing 0.2-mM imidazole and 8% BME) and subsequently boiled for 5 min.

Input samples that had been set aside were lysed by resuspending the P in EZ buffer (0.06 M Tris HCl pH 6.8, 10% glycerol, 2% SDS, 5% BME) and boiled for 10 min. Bradford assays were conducted to determine protein concentration, and 10 μg of each sample was resuspended in 2× sample buffer (containing 0.2 mM imidazole and 8% BME) and further boiled for another 5 minutes. Ten micrograms of the input sample and 30 μL of each pull-down sample were loaded onto 10% denaturing gels and subjected to immunoblot analyses.

### FLAG pull-down assays

Overnight cultures of *RPT5* and *rpt5–P76A* strains expressing endogenously tagged Nas2–6xGly–3xFLAG were diluted to absorbance at 600 nm = 0.2 and grown in 100-ml YPD to the midexponential phase at 30 °C, washed with ice-cold water, and subsequently frozen in liquid nitrogen and stored at −80 °C. Frozen cells in liquid N_2_ were ground using a mortar and pestle until a fine powder formed. Cell powders were incubated in buffer F (50 mM Tris HCl pH 7.5, 150 mM NaCl, 10% glycerol, 5 mM MgCl_2_, 5 mM ATP) on ice and vortexed intermittently for 10 min. Samples were then centrifuged at 20,000*g* for 10 min to remove cell debris. The resulting S was transferred into a fresh tube, and protein concentrations were determined *via* the BCA assay.

T was normalized to 4.94 mg in 1.2-ml final volume for each sample. Hundred microliters T was set aside as the input. The remaining samples were incubated with 100-μL anti-FLAG resin on a rotating mixer at 4 °C for 2 h. The anti-FLAG resin was centrifuged, and the S was removed. FLAG resin was washed thrice with 1-ml buffer F. Bound proteins were subsequently eluted with 300-μL buffer F containing 200 μg/ml 3xFLAG peptide. Input samples and FLAG eluates were brought to 1× concentration sample buffer containing 1% BME and heated at 100 °C for 5 min. Eight microliters input and 30-μL pull-down eluate were loaded onto 12% denaturing gels and subjected to immunoblot analyses.

## Data availability

All data are contained within this article.

## Supporting information

This article contains supporting information (42, 43, 44, 45, 46, 47).

## Conflict of interest

The authors declare that they have no conflicts of interest with the contents of the article.
