# Supplementary material for: Conserved proline residues in the coiled coil–OB domain linkers of Rpt proteins facilitate eukaryotic proteasome base assembly
Source: J Biol Chem. 2021 Apr 14;296:100660. doi: 10.1016/j.jbc.2021.100660 (PMC8134078; doi:10.1016/j.jbc.2021.100660)
Supplement: Figures S1 to S10 and Tables S1 to S3 [file mmc1.pdf]

SUPPORTING INFORMATION

Conserved Proline Residues in the Coiled Coil-OB Domain Linkers of Rpt Proteins Facilitate Eukaryotic Proteasome Base Assembly

Chin Leng Cheng[1], Michael K Wong[1], Yanjie Li[1], Mark Hochstrasser[1,2]*

[1] Department of Molecular Biophysics and Biochemistry, Yale University, New Haven, Connecticut, USA

[2] Department of Molecular, Cellular, and Developmental Biology, Yale University, New Haven, Connecticut, USA

* Corresponding author: Mark Hochstrasser

E-mail: mark.hochstrasser@yale.edu

Figure S1. Genetic interactions between *rpt* P-to-A mutations and different base and CP assembly chaperone gene deletions

Figure S2. Test of genetic interactions between *adc17*Δ and *rpt2,5PA*

Figure S3. Effects of combining *rpn4*Δ and *rpt* P-to-A mutations

Figure S4. Effects of *HSM3* deletion on accumulation of Rpt2-containing subcomplexes

Figure S5. Growth analysis of *rpt* double P-to-A mutants

Figure S6. Analysis of anti-FLAG co-immunoprecipitation of Rpt4 and Rpt5 in *RPT5* and *rpt5-P76A* cells expressing Nas2-6xGly-3xFLAG

Figure S7. Growth analysis of *pre9Δ* and *sem1Δ* assembly mutants carrying an inactive *not4-L35A* ubiquitin ligase gene

Figure S8. Analysis of aggregation and ubiquitination of proteasome base subunits in *rpt2,5PA* cells

Figure S9. Analysis of aggregation of base assembly chaperones in *rpt2,5PA* and *rpt2,5PA hsp42*Δ cells

Figure S10. Growth analysis of select base, CP, and lid assembly mutants with the chromosomal *HSP42* gene deleted

Table S1. List of the eukaryotic species used for phylogenetic analyses in Figure 2A

Table S2. List of yeast strains used in this study

Table S3. List of plasmids used in this study

Supporting Information Figure Legends

Figure S1. Growth assays evaluating potential synthetic interactions between single *rpt* P-to-A mutations and different base and CP assembly chaperone gene deletions. Cells were grown on YPD plates at 36°C.

Figure S2. Deletion of *ADC17* displays an extremely weak synthetic interaction with *rpt2,5PA*

(A) No synthetic growth defects observed between *adc17*Δ and *rpt1-P76A*, *rpt2-P103A*, *rpt3-P96A,* or *rpt5-P76A.*

(B) A very slight synthetic growth defect observed between *adc17*Δ and *rpt2,5PA.*

Figure S3. Genetic interactions between *rpn4*Δ and *rpt* P-to-A mutations

(A) No synthetic growth defects observed between *rpn4*Δ and *rpt1-P76A*, *rpt2-P103A*, or *rpt3-P96A.* Serially diluted cultures were spotted on plates as in Figure 2B.

(B) Immunoblot analyses of yeast proteasome complexes in *rpt5-P76A* cells with and without deletion of *RPN4*.

Figure S4. Anti-FLAG immunoprecipitation of Nas2-6xGly-3xFLAG shows similar ratios of co-precipitated Rpt4 and Rpt5 levels in *RPT5* and *rpt5-P76A* strains

Figure S5. No significant growth defects are observed in the indicated *rpt* double P-to-A mutants. Serially diluted cultures were spotted on plates as in Figure 2B.

Figure S6. Deletion of *HSM3* in *RPT2* and *rpt2-P103A* strains leads to accumulation of Rpt2-containing subcomplexes as also seen in *rpt2,5PA* cells

Figure S7. The *not4-L35A* ubiquitin ligase mutation does not suppress the growth defects of the tested CP (*pre9Δ*) and RP (*sem1Δ*) assembly mutants

Figure S8. Analysis of aggregation and ubiquitination of select proteasome base subunits in *rpt2,5PA* mutant cells

(A) Aggregation assays for *rpt2,5PA* and WT cells. The data suggest aggregation of the base subunit Rpt3 increases in the mutant, whereas base subunit Rpn2 aggregation is unaffected. Bulk Rpt4 steady-state levels are notably decreased in *rpt2,5PA* when cells are grown to saturation. CP subunit α4 aggregation is not obviously affected by *rpt2,5PA*, and aggregation of lid subunit Rpn12 is also unaffected.

(B) Purification of bulk His_6-ubiquitin conjugates reveals comparable Rpt3 ubiquitination in both WT and *rpt2,5PA* strains at 37˚C, as in Figure 5B. Arrowhead denotes unmodified Rpt3 bands. *, Ubiquitinated Rpt3 species.

Figure S9. Analysis of aggregation of base assembly chaperones in WT and *rpt2,5PA* strains with or without *HSP42*

Yeast strains were grown in YPD at 37°C to mid-exponential phase. Little or no difference between the strains was evident.

Figure S10. *HSP42* deletion suppresses growth defects of additional base (*cim3-1* and *rpt4-G106D*) and CP (*pre9Δ*) mutants but not that of a *sem1Δ* (lid) mutant

Table S1. List of the eukaryotic species used for phylogenetic analyses

	**Supergroup**	**Group**	**Species**	**Abbreviation**
1	Opisthokonts	Ascomycetes	*Saccharomyces cerevisiae*	*sc*
2	Opisthokonts	Animals	*Homo sapiens*	*hs*
3	Amoebozoa	Dictyostelids	*Dictyostelium purpureum*	*dp*
4	Amoebozoa	Dictyostelids	*Dictyostelium discoideum*	*dd*
5	Excavates	Diplomonads	*Giardia lamblia*	*gl*
6	Excavates	Heterolobosea	*Naegleria gruberi*	*ng*
7	Excavates	Trichomonads	*Trichomonas vaginalis*	*tv*
8	Excavates	Kinetoplastids	*Trypanosoma brucei*	*tb*
9	Excavates	Kinetoplastids	*Leishmania infantum*	*li*
10	Archaeplastida	Land plants	*Arabidopsis thaliana*	*at*
11	Archaeplastida	Chlorophytes	*Chlamydomonas reinhardtii*	*cr*
12	Archaeplastida	Floridiophytes	*Chondrus crispus*	*cc*
13	Archaeplastida	Cyanidiophytes	*Cyanidioschyzon merole*	*cm*
14	Hacrobia	Cryptomonads	*Cryptomonas paramecium*	*cp*
15	Hacrobia	Cryptomonads	*Guillardia theta*	*gt*
16	Hacrobia	Cryptomonads	*Hemiselmis andersenii*	*ha*
17	SAR	Apicomplexa	*Plasmodium falciparum*	*pf*
18	SAR	Apicomplexa	*Toxoplasma gondii*	*tg*
19	SAR	Oomycetes	*Aphanomyces invadans*	*ai*
20	SAR	Oomycetes	*Phytophthora parasitica*	*pp*
21	SAR	Diatoms	*Thalassiosira pseudonana*	*tp*
22	SAR	Ciliates	*Ichthyophthirius multifiliis*	*im*

Table S2: List of yeast strains used in this study

Strain	Genotype	Source and Reference
MHY500	*MATa his3-Δ200 leu2-3,112 ura3-52 lys2-801 trp1-1 gal2*	(1)
MHY1069	*MATα his3-Δ200 leu2-3,112 ura3-52 lys2-801 trp1-1 gal2 pre9Δ::HIS3*	(2)
MHY2836	*MATa ura3-52 lys2-801 ade2-101 trp1-Δ63 his3-Δ200 leu2-Δ1*	(3)
MHY4401	*MATa ura3-52 lys2-801 ade2-101 trp1-Δ63 his3-Δ200 leu2-Δ1 rpt4-G106D*	(4)
MHY4464	*MATa ura3-52 lys2-801 ade2-101 trp1-Δ63 his3-Δ200 leu2-Δ1 rpt6(cim3-1)*	(4)
MHY4785	*MATa his3-Δ200 leu2-3,112 ura3-52 lys2-801 trp1-1 gal2 sem1Δ::HIS3*	(5)
MHY5659	*MATa his3-Δ200 leu2-3,112 ura3-52 lys2-801 trp1-1 gal2 rpt1Δ::HIS3* [pFL44CIM5 (RPT1)]	(6)
MHY5660	*MATα his3-Δ200 leu2-3,112 ura3-52 lys2-801 trp1-1 gal2 rpt2Δ::HIS3* [pRS316RPT2]	(6)
MHY5661	*MATα his3-Δ200 leu2-3,112 ura3-52 lys2-801 trp1-1 gal2 rpt5Δ::HIS3* [YCplac33RPT5]	(6)
MHY5685	*MATα his3-Δ200 leu2-3,112 ura3-52 lys2-801 trp1-1 gal2rpt1Δ::HIS3 rpt2Δ::HIS3* [pFL44CIM5 + pRS316RPT2]	(6)
MHY5745	*MATα his3-Δ200 leu2-3,112 ura3-52 lys2-801 trp1-1 gal2 rpt3Δ::HIS3* [YCplac33RPT3]	(6)
MHY5754	*MATα his3-Δ200 leu2-3,112 ura3-52 lys2-801 trp1-1 gal2 rpt1Δ::HIS3 rpt5Δ::HIS3* [pFL44CIM5 + YCplac33RPT5]	(6)
MHY5776	*MATα his3-Δ200 leu2-3,112 ura3-52 lys2-801 trp1-1 gal2 rpt1Δ::HIS3 rpt3Δ::HIS3* [YCplac33RPT1 + YCplac33RPT3]	(6)
MHY6008	*MATa his3-Δ200 leu2-3,112 ura3-52 lys2-801 trp1-1 gal2 rpt1Δ::HIS3 hsm3Δ::kanMX* [pFL44CIM5 (RPT1)]	This study
MHY6009	*MATα his3-Δ200 leu2-3,112 ura3-52 lys2-801 trp1-1 gal2 rpt2Δ::HIS3 hsm3Δ::kanMX* [pRS316RPT2]	This study
MHY6010	*MATα his3-Δ200 leu2-3,112 ura3-52 lys2-801 trp1-1 gal2 rpt2Δ::HIS3 nas2Δ::kanMX* [pRS316RPT2]	This study
MHY6011	*MATα his3-Δ200 leu2-3,112 ura3-52 lys2-801 trp1-1 gal2 rpt2Δ::HIS3 nas6Δ::kanMX* [pRS316RPT2]	This study
MHY6012	*MATα his3-Δ200 leu2-3,112 ura3-52 lys2-801 trp1-1 gal2 rpt2Δ::HIS3 rpn14Δ::kanMX* [pRS316RPT2]	This study
MHY6071	*MATα his3-Δ200 leu2-3,112 ura3-52 lys2-801 trp1-1 gal2 rpt3Δ::HIS3 hsm3Δ::kanMX* [YCplac33RPT3]	This study
MHY6072	*MATα his3-Δ200 leu2-3,112 ura3-52 lys2-801 trp1-1 gal2 rpt3Δ::HIS3 nas6Δ::kanMX* [YCplac33RPT3]	This study
MHY6074	*MATα his3-Δ200 leu2-3,112 ura3-52 lys2-801 trp1-1 gal2 rpt3Δ::HIS3 rpn14Δ::kanMX* [YCplac33RPT3]	This study
MHY6076	*MATα his3-Δ200 leu2-3,112 ura3-52 lys2-801 trp1-1 gal2 rpt3Δ::HIS3 nas2Δ::kanMX* [YCplac33RPT3]	This study
MHY6077	*MATα his3-Δ200 leu2-3,112 ura3-52 lys2-801 trp1-1 gal2 rpt5Δ::HIS3 hsm3Δ::kanMX* [YCplac33RPT5]	This study
MHY6078	*MATa his3-Δ200 leu2-3,112 ura3-52 lys2-801 trp1-1 gal2 rpt5Δ::HIS3 nas6Δ::kanMX* [YCplac33RPT5]	This study

MHY6080	*MATα his3-Δ200 leu2-3,112 ura3-52 lys2-801 trp1-1 gal2 rpt5Δ::HIS3 rpn14Δ::kanMX* [YCplac33RPT5]	This study
MHY6082	*MATa his3-Δ200 leu2-3,112 ura3-52 lys2-801 trp1-1 gal2 rpt5Δ::HIS3 nas2Δ::kanMX* [YCplac33RPT5]	This study
MHY6211	*MATα his3-Δ200 leu2-3,112 ura3-52 lys2-801 trp1-1 gal2 rpt2Δ::HIS3 rpt3Δ::HIS3* [pRS316RPT2 + YCplac33RPT3]	This study
MHY6213	*MATa his3-Δ200 leu2-3,112 ura3-52 lys2-801 trp1-1 gal2 rpt2Δ::HIS3 rpt5Δ::HIS3* [pRS316RPT2 + YCplac33RPT5]	This study
MHY6214	*MATa his3-Δ200 leu2-3,112 ura3-52 lys2-801 trp1-1 gal2 rpt3Δ::HIS3 rpt5Δ::HIS3 HIS3* [YCplac33RPT3 + YCplac33RPT3]	This study
MHY9685	*MATa his3-Δ200 leu2-3,112 ura3-52 lys2-801 trp1-1 gal2 rpt5Δ::HIS3 rpn4Δ::kanMX* [YCplac33RPT5]	This study
MHY9716	*MATα his3-Δ200 leu2-3,112 ura3-52 lys2-801 trp1-1 gal2 rpt2Δ::HIS3 rpn4Δ::kanMX* [pRS316RPT2]	This study
MHY9728	*MATα his3-Δ200 leu2-3,112 ura3-52 lys2-801 trp1-1 gal2 rpt3Δ::HIS3 rpn4Δ::kanMX* [YCplac33RPT3]	This study
MHY9730	*MATα his3-Δ200 leu2-3,112 ura3-52 lys2-801 trp1-1 gal2 rpt1Δ::HIS3 rpn4Δ::kanMX* [pFL44CIM5 (RPT1)]	This study
MHY10300	*MATα his3-Δ200 leu2-3,112 ura3-52 lys2-801 trp1-1 gal2 rpt1Δ::HIS3 adc17Δ::hphMX* [pFL44CIM5 (RPT1)]	This study
MHY10302	*MATa his3-Δ200 leu2-3,112 ura3-52 lys2-801 trp1-1 gal2 rpt2Δ::HIS3 adc17Δ::hphMX* [pRS316RPT2]	This study
MHY10304	*MATα his3-Δ200 leu2-3,112 ura3-52 lys2-801 trp1-1 gal2 rpt3Δ::HIS3 adc17Δ::hphMX* [YCplac33RPT3]	This study
MHY10306	*MATα his3-Δ200 leu2-3,112 ura3-52 lys2-801 trp1-1 gal2 rpt5Δ::HIS3 adc17Δ::hphMX* [YCplac33RPT5]	This study
MHY10404	*MATα his3-Δ200 leu2-3,112 ura3-52 lys2-801 trp1-1 gal2 hsp42Δ::hphMX*	This study
MHY10468	*MATα his3-Δ200 leu2-3,112 ura3-52 lys2-801 trp1-1 gal2 rpt2Δ::HIS3 pba1Δ::kanMX* [pRS316RPT2]	This study
MHY10470	*MATα his3-Δ200 leu2-3,112 ura3-52 lys2-801 trp1-1 gal2 rpt5Δ::HIS3 pba1Δ::kanMX* [YCplac33RPT5]	This study
MHY10471	*MATα his3-Δ200 leu2-3,112 ura3-52 lys2-801 trp1-1 gal2 rpt2Δ::HIS3 pba4Δ::hphMX* [pRS316RPT2]	This study
MHY10472	*MATα his3-Δ200 leu2-3,112 ura3-52 lys2-801 trp1-1 gal2 rpt5Δ::HIS3 pba4Δ::hphMX* [YCplac33RPT5]	This study
CLC123	*MATα his3-Δ200 leu2-3,112 ura3-52 lys2-801 trp1-1 gal2 rpt5Δ::HIS3 NAS2-6xGly-3xFLAG::kanMX* [YCplac33RPT5]	This study
CLC148	*MATα his3-Δ200 leu2-3,112 ura3-52 lys2-801 trp1-1 gal2 not4Δ::natMX*	This study
CLC164	*MATa ura3-52 lys2-801 ade2-101 trp1-Δ63 his3-Δ200 leu2-Δ1 hsp42Δ::hphMX*	This study
CLC166	*MATα his3-Δ200 leu2-3,112 ura3-52 lys2-801 trp1-1 gal2 rpt2Δ::HIS3 rpt5Δ::HIS3 hsp42Δ::hphMX* [pRS316RPT2 + YCplac33RPT5]	This study
CLC173	*MATα his3-Δ200 leu2-3,112 ura3-52 lys2-801 trp1-1 gal2 pre9Δ::HIS3 not4Δ::natMX*	This study

CLC182	*MATα his3-Δ200 leu2-3,112 ura3-52 lys2-801 trp1-1 gal2 sem1Δ::HIS3 not4Δ::natMX*	This study
CLC208	*MATa ura3-52 lys2-801 ade2-101 trp1-Δ63 his3-Δ200 leu2-Δ1 rpt6(cim3-1) hsp42Δ::hphMX*	This study
CLC209	*MATa ura3-52 lys2-801 ade2-101 trp1-Δ63 his3-Δ200 leu2-Δ1 rpt4-G106D hsp42Δ::hphMX*	This study
CLC211	*MATa his3-Δ200 leu2-3,112 ura3-52 lys2-801 trp1-1 gal2 pre9Δ::HIS3 hsp42Δ::hphMX*	This study
CLC214	*MATα his3-Δ200 leu2-3,112 ura3-52 lys2-801 trp1-1 gal2 sem1Δ::HIS3 hsp42Δ::hphMX*	This study
CLC287	*MATa his3-Δ200 leu2-3,112 ura3-52 lys2-801 trp1-1 gal2 rpt2Δ::HIS3 rpt5Δ::HIS3 not4Δ::kanMX* [YCplac111-*RPT2* + YCplac22-*RPT5* + pRS317-*NOT4*]	This study
CLC288	*MATa his3-Δ200 leu2-3,112 ura3-52 lys2-801 trp1-1 gal2 rpt2Δ::HIS3 rpt5Δ::HIS3 not4Δ::kanMX* [YCplac111-*RPT2* + YCplac22-*RPT5* + pRS317-*not4-L35A*]	This study
CLC289	*MATa his3-Δ200 leu2-3,112 ura3-52 lys2-801 trp1-1 gal2 rpt2Δ::HIS3 rpt5Δ::HIS3 not4Δ::kanMX* [YCplac111-*rpt2-P103A* + YCplac22-*rpt5-P76A* + pRS317-*NOT4*]	This study
CLC290	*MATa his3-Δ200 leu2-3,112 ura3-52 lys2-801 trp1-1 gal2 rpt2Δ::HIS3 rpt5Δ::HIS3 not4Δ::kanMX* [YCplac111-*rpt2-P103A* + YCplac22-*rpt5-P76A* + pRS317-*not4-L35A*]	This study
CLC560	*MATα his3-Δ200 leu2-3,112 ura3-52 lys2-801 trp1-1 gal2 rpt2Δ::HIS3 rpt5Δ::HIS3 hsp42Δ::hphMX not4Δ::kanMX* [YCplac111-*RPT2* + YCplac22-*RPT5* + pRS317-*NOT4*]	This study
CLC561	*MATα his3-Δ200 leu2-3,112 ura3-52 lys2-801 trp1-1 gal2 rpt2Δ::HIS3 rpt5Δ::HIS3 hsp42Δ::hphMX not4Δ::kanMX* [YCplac111-*RPT2* + YCplac22-*RPT5* + pRS317-*not4-L35A*]	This study
CLC562	*MATα his3-Δ200 leu2-3,112 ura3-52 lys2-801 trp1-1 gal2 rpt2Δ::HIS3 rpt5Δ::HIS3 hsp42Δ::hphMX not4Δ::kanMX* [YCplac111-*RPT2* + YCplac22-*RPT5* + pRS317-*not4-L35A*]	This study
CLC563	*MATα his3-Δ200 leu2-3,112 ura3-52 lys2-801 trp1-1 gal2 rpt2Δ::HIS3 rpt5Δ::HIS3 hsp42Δ::hphMX not4Δ::kanMX* [YCplac111-*rpt2-P103A* + YCplac22-*rpt5-P76A* + pRS317-*not4-L35A*]	This study
CLC565	*MATa his3-Δ200 leu2-3,112 ura3-52 lys2-801 trp1-1 gal2 rpt2Δ::HIS3 rpt5Δ::HIS3 adc17Δ::hphMX* [pRS316RPT2 + YCplac33RPT5]	This study

Table S3: List of plasmids used in this study

**Plasmids**	**Source**
pFL44-*CIM5 (RPT1)*	(6)
pRS316-*RPT2*	(6)
YCplac33-*RPT3*	(6)
YCplac33-*RPT5*	(6)
pRS314-*RPT1*	(6)
pRS314-*rpt1-P96A*	This study

YCplac111-*RPT2*	(6)
YCplac111-*rpt2-P103A*	This study
YCplac111-*RPT3*	(6)
YCplac111-*rpt3-P93A*	This study
YCplac111-*RPT5*	(6)
YCplac111-*rpt5-P76A*	This study
YCplac22-*RPT5*	(6)
YCplac22-*rpt5-P76A*	This study
pRS314-*RPT2*	This study
pRS314-*rpt2-P103A*	This study
YCplac22-*rpt5-L75A*	This study
YCplac22-*rpt5-Y77A*	This study
YCplac111-*rpt2-N102A*	This study
YCplac111-*rpt2-L104A*	This study
pRS317-*NOT4*	This study
pRS317-*not4-L35A*	This study
pET15b-6His-Rpt5	This study
pET15b-6His-rpt5(P76A)	This study
pUB175	D. Finley
pUB221	D. Finley

References

1. Chen, P., Johnson, P., Sommer, T., Jentsch, S., and Hochstrasser, M. (1993) Multiple ubiquitin-conjugating enzymes participate in the in vivo degradation of the yeast MAT alpha 2 repressor. *Cell* **74**, 357-369
2. Velichutina, I., Connerly, P. L., Arendt, C. S., Li, X., and Hochstrasser, M. (2004) Plasticity in eucaryotic 20S proteasome ring assembly revealed by a subunit deletion in yeast. *Embo j* **23**, 500-510
3. Sikorski, R. S., and Hieter, P. (1989) A system of shuttle vectors and yeast host strains designed for efficient manipulation of DNA in Saccharomyces cerevisiae. *Genetics* **122**, 19-27
4. Funakoshi, M., Tomko, R. J., Jr., Kobayashi, H., and Hochstrasser, M. (2009) Multiple assembly chaperones govern biogenesis of the proteasome regulatory particle base. *Cell* **137**, 887-899
5. Tomko, R. J., Jr., and Hochstrasser, M. (2011) Incorporation of the Rpn12 subunit couples completion of proteasome regulatory particle lid assembly to lid-base joining. *Mol Cell* **44**, 907-917
6. Tomko, R. J., Jr., Funakoshi, M., Schneider, K., Wang, J., and Hochstrasser, M. (2010) Heterohexameric ring arrangement of the eukaryotic proteasomal ATPases: implications for proteasome structure and assembly. *Mol Cell* **38**, 393-403

FIGURE S1

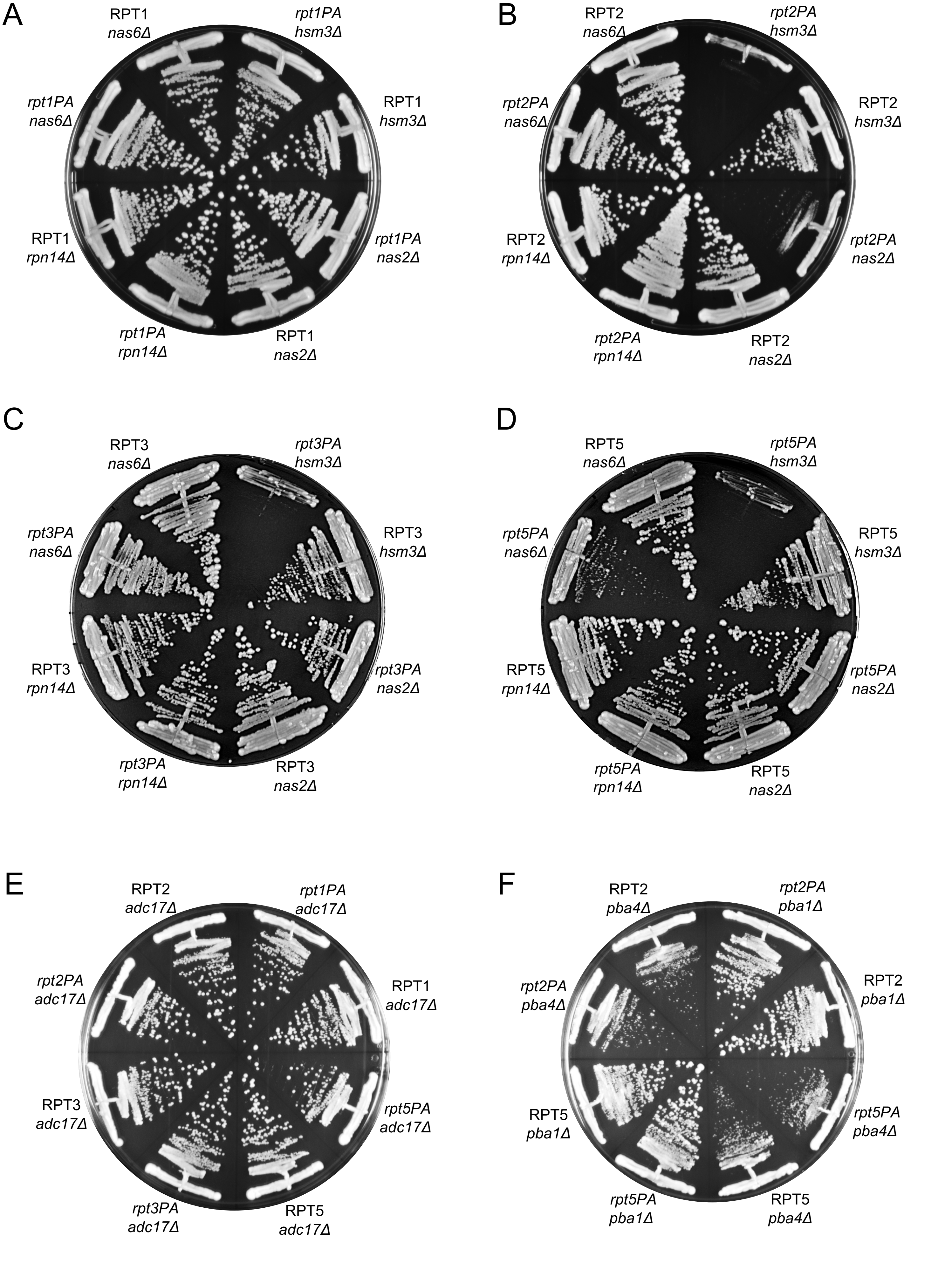
A
RPT1 nas6Δ
rpt1PA hsm3Δ
rpt1PA nas6Δ
RPT1 hsm3Δ
RPT1 rpn14Δ
rpt1PA nas2Δ
rpt1PA rpn14Δ
RPT1 nas2Δ
B
RPT2 nas6Δ
rpt2PA hsm3Δ
rpt2PA nas6Δ
RPT2 hsm3Δ
RPT2 rpn14Δ
rpt2PA nas2Δ
rpt2PA rpn14Δ
RPT2 nas2Δ
C
RPT3 nas6Δ
rpt3PA hsm3Δ
rpt3PA nas6Δ
RPT3 hsm3Δ
RPT3 rpn14Δ
rpt3PA nas2Δ
rpt3PA rpn14Δ
RPT3 nas2Δ
D
RPT5 nas6Δ
rpt5PA hsm3Δ
rpt5PA nas6Δ
RPT5 hsm3Δ
RPT5 rpn14Δ
rpt5PA nas2Δ
rpt5PA rpn14Δ
RPT5 nas2Δ
E
RPT2 adc17Δ
rpt1PA adc17Δ
rpt2PA adc17Δ
RPT1 adc17Δ
RPT3 adc17Δ
rpt5PA adc17Δ
rpt3PA adc17Δ
RPT5 adc17Δ
F
RPT2 pba4Δ
rpt2PA pba1Δ
rpt2PA pba4Δ
RPT2 pba1Δ
RPT5 pba1Δ
rpt5PA pba4Δ
rpt5PA pba1Δ
RPT5 pba4Δ

FIGURE S2

A

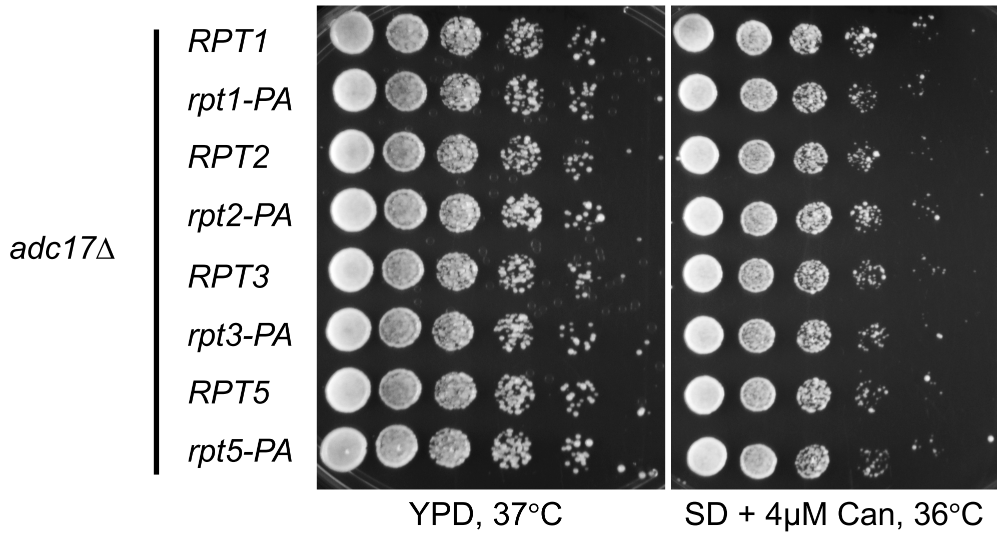
adc17Δ
RPT1
rpt1-PA
RPT2
rpt2-PA
RPT3
rpt3-PA
RPT5
rpt5-PA
YPD, 37°C
SD + 4μM Can, 36°C

B

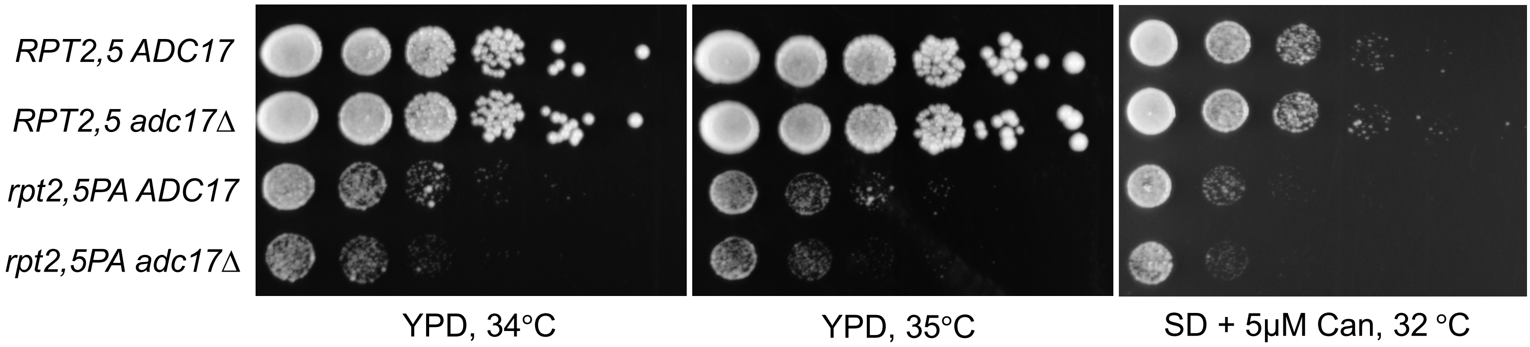
RPT2,5 ADC17
RPT2,5 adc17Δ
rpt2,5PA ADC17
rpt2,5PA adc17Δ
YPD, 34°C
YPD, 35°C
SD + 5μM Can, 32 °C

FIGURE S3

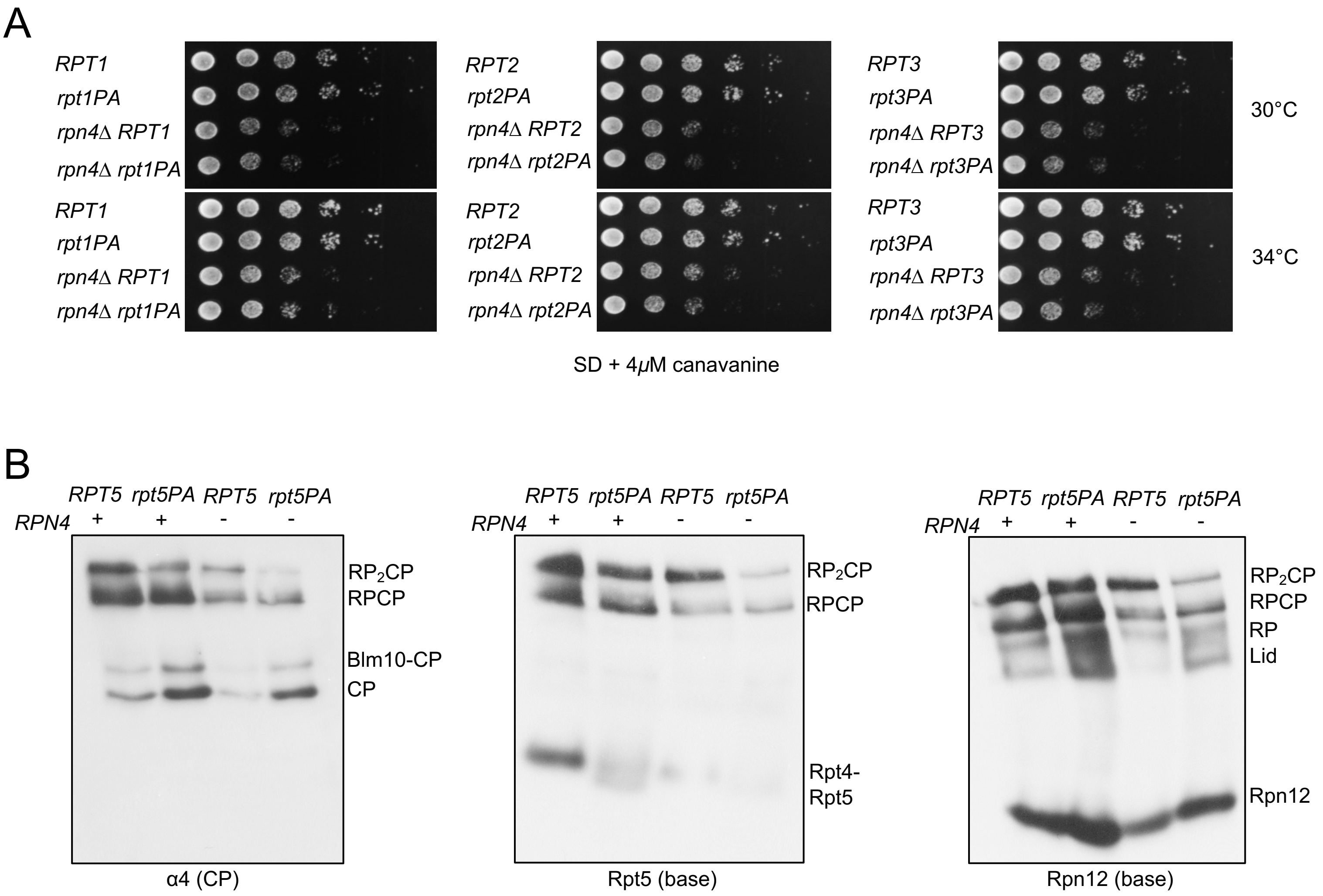
A
RPT1
rpt1PA
rpn4Δ RPT1
rpn4Δ rpt1PA
RPT2
rpt2PA
rpn4Δ RPT2
rpn4Δ rpt2PA
RPT3
rpt3PA
rpn4Δ RPT3
rpn4Δ rpt3PA
30°C
34°C
SD + 4μM canavanine
B
RPT5 rpt5PA RPT5 rpt5PA
RPN4 + + - -
RP2CP
RPCP
Blm10-CP
CP
α4 (CP)
Rpt4-Rpt5
Rpt5 (base)
RP
Lid
Rpn12
Rpn12 (base)

FIGURE S4

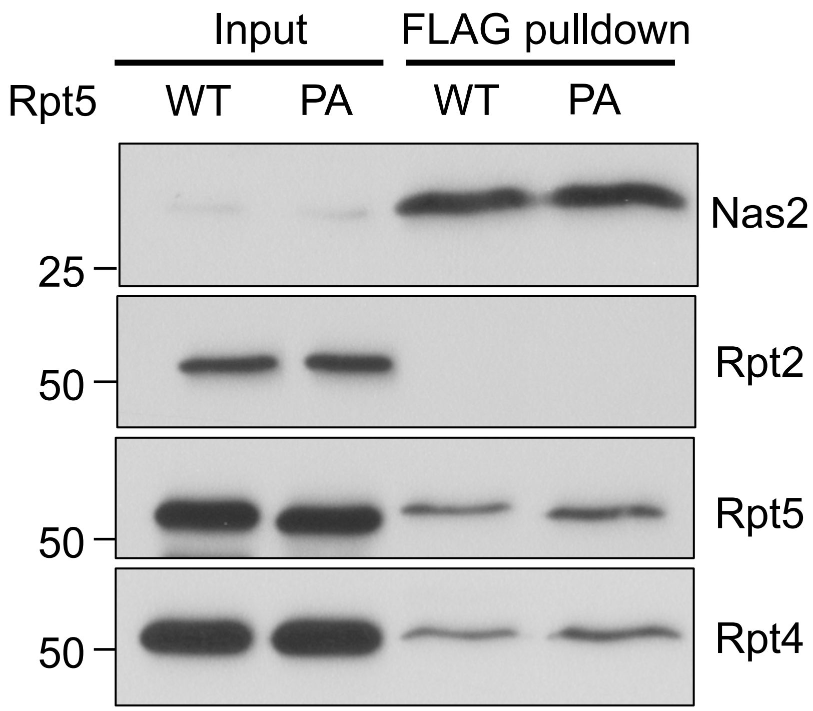
Input
FLAG pulldown
Rpt5
WT
PA
WT
PA
Nas2
25
Rpt2
50
Rpt5
50
Rpt4
50

FIGURE S5

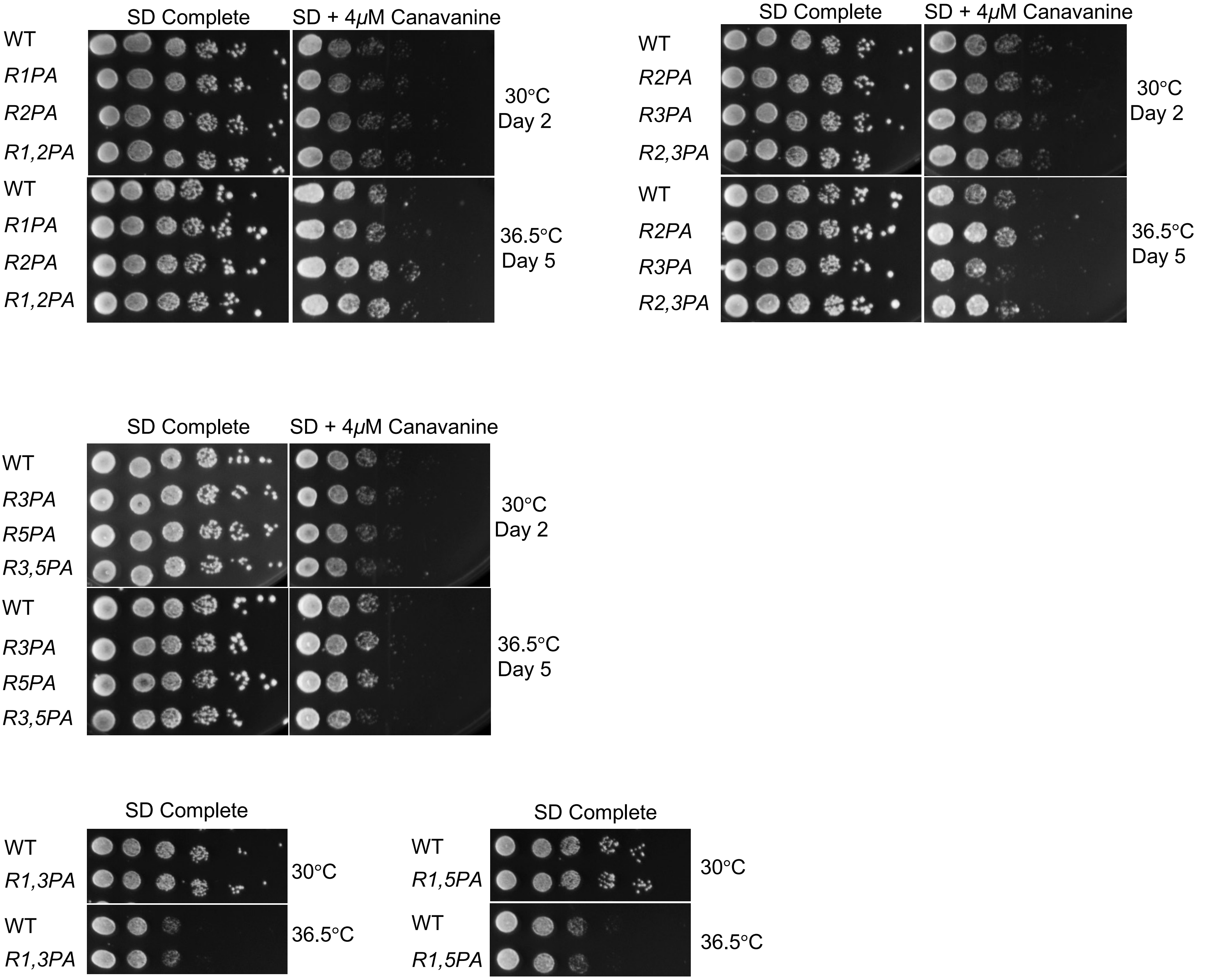
SD Complete
SD + 4μM Canavanine
WT
R1PA
R2PA
R1,2PA
30°C
Day 2
WT
R1PA
R2PA
R1,2PA
36.5°C
Day 5
SD Complete
SD + 4μM Canavanine
WT
R2PA
R3PA
R2,3PA
30°C
Day 2
WT
R2PA
R3PA
R2,3PA
36.5°C
Day 5
SD Complete
SD + 4μM Canavanine
WT
R3PA
R5PA
R3,5PA
30°C
Day 2
WT
R3PA
R5PA
R3,5PA
36.5°C
Day 5
SD Complete
WT
R1,3PA
30°C
WT
R1,3PA
36.5°C
SD Complete
WT
R1,5PA
30°C
WT
R1,5PA
36.5°C

FIGURE S6

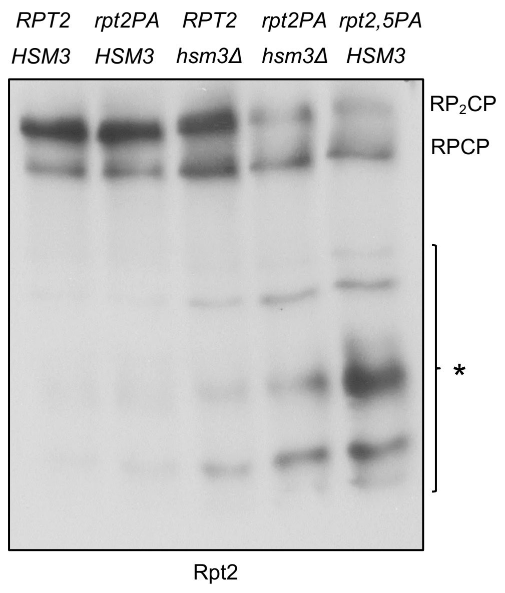
RPT2
HSM3
rpt2PA
HSM3
RPT2
hsm3Δ
rpt2PA
hsm3Δ
rpt2,5PA
HSM3
RP2CP
RPCP
*
Rpt2

FIGURE S7

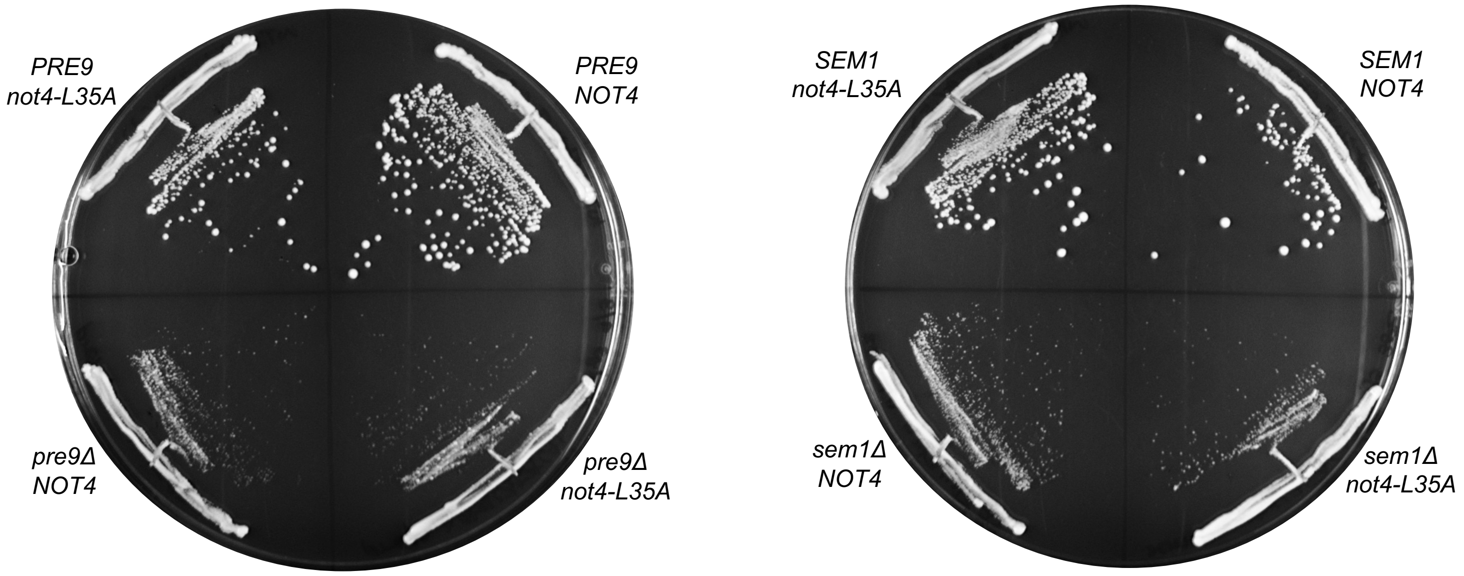

SD-Lys, 36°C SD-Lys, 36°C

FIGURE S8

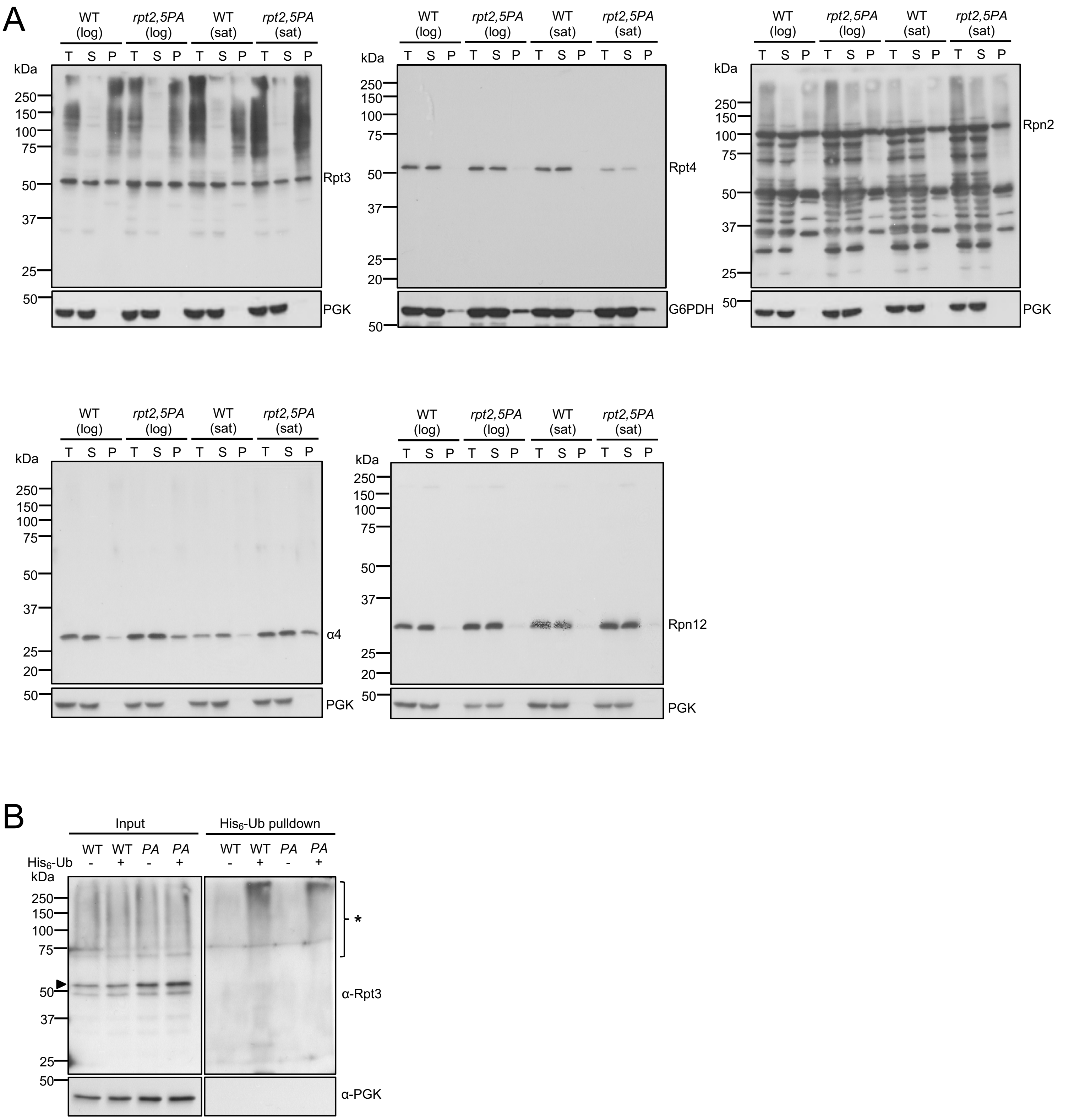
A
WT (log)
rpt2,5PA (log)
WT (sat)
rpt2,5PA (sat)
T S P T S P T S P T S P
kDa
250
150
100
75
50
37
25
20
Rpt3
PGK
Rpt4
G6PDH
Rpn2
α4
Rpn12
B
Input
His6-Ub pulldown
WT WT PA PA
His6-Ub - + - +
*
α-Rpt3
α-PGK

FIGURE S9

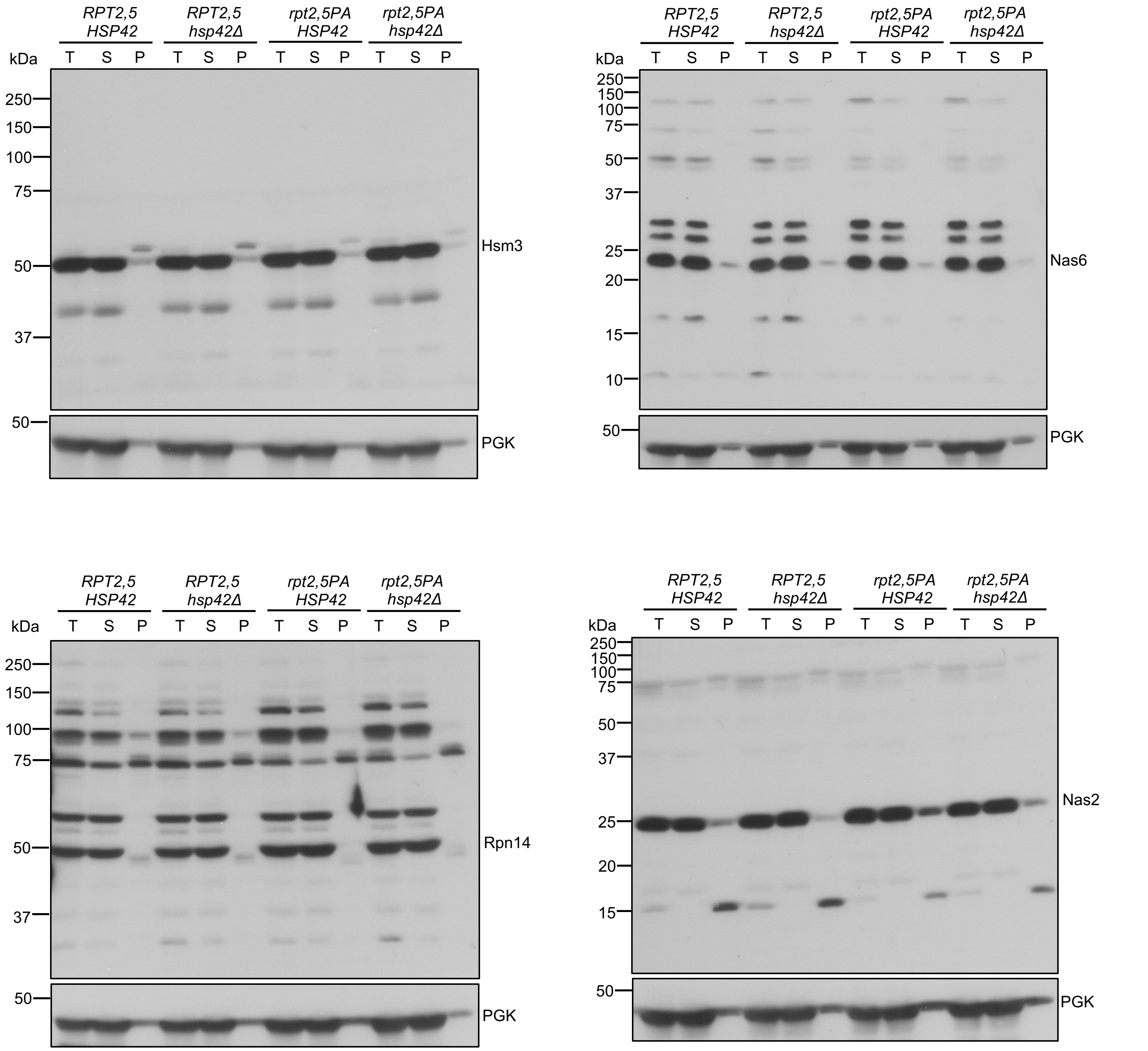
RPT2,5 HSP42
RPT2,5 hsp42Δ
rpt2,5PA HSP42
rpt2,5PA hsp42Δ
kDa
T S P T S P T S P T S P
250
150
100
75
50
37
Hsm3
PGK
25
20
15
10
Nas6
Rpn14
Nas2

FIGURE S10

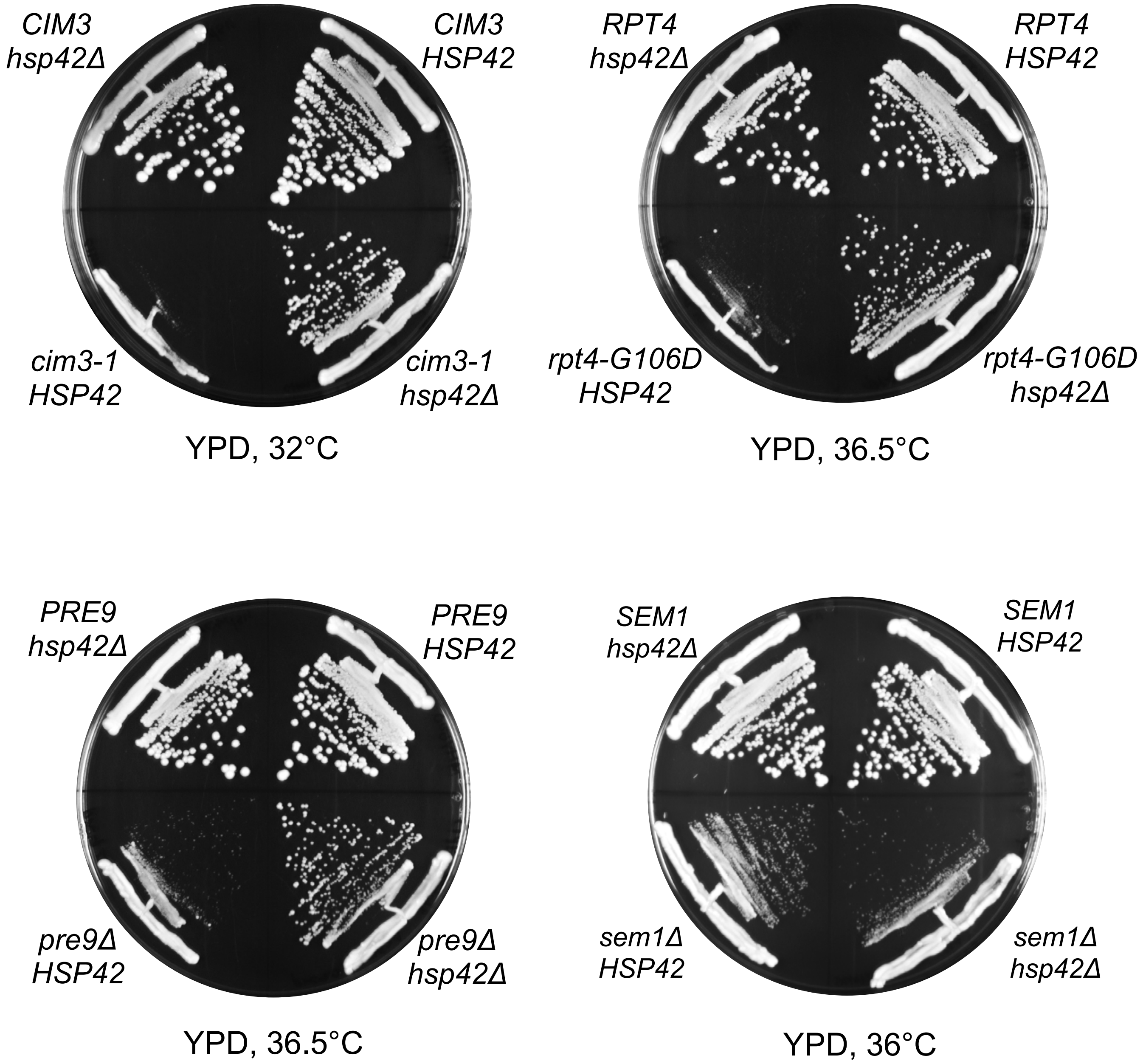

CIM3 hsp42Δ
CIM3 HSP42
cim3-1 HSP42
cim3-1 hsp42Δ
YPD, 32°C
RPT4 hsp42Δ
RPT4 HSP42
rpt4-G106D HSP42
rpt4-G106D hsp42Δ
YPD, 36.5°C
PRE9 hsp42Δ
PRE9 HSP42
pre9Δ HSP42
pre9Δ hsp42Δ
YPD, 36.5°C
SEM1 hsp42Δ
SEM1 HSP42
sem1Δ HSP42
sem1Δ hsp42Δ
YPD, 36°C
